# Exomeres From Adventitial Fibroblasts of Spontaneously Hypertensive Rats Promote Vascular Remodelling via Transferring Osteopontin

**DOI:** 10.1002/jev2.70146

**Published:** 2025-08-06

**Authors:** Jing‐Xiao Wang, Xiao‐Yu Xu, Hong‐Ke Dong, Yi‐Ming Wang, Min Dai, Bing Zhou, Yue‐Hua Li, Guo‐Qing Zhu, Xiao‐Qing Xiong

**Affiliations:** ^1^ Key Laboratory of Targeted Intervention of Cardiovascular Disease Collaborative Innovation Center for Cardiovascular Disease Translational Medicine Department of Physiology Nanjing Medical University Nanjing Jiangsu China; ^2^ Department of Pathology Yijishan Hospital The First Affiliated Hospital of Wannan Medical College Wuhu Anhui China; ^3^ Department of Pathophysiology Nanjing Medical University Nanjing Jiangsu China

**Keywords:** exomere, hypertension, osteopontin, vascular adventitial fibroblast, vascular remodelling, vascular smooth muscle cell

## Abstract

Vascular adventitial fibroblasts (VAFs) contribute to vascular remodelling in hypertension. However, the mechanisms by which VAFs regulate vascular smooth muscle cells (VSMCs) in vascular remodelling are not well known. Here we report the crucial roles of extracellular nanoparticles exomeres (EMs) derived from VAFs in promoting VSMCs proliferation, migration and vascular remodelling in normotensive Wistar‐Kyoto rats (WKY) and spontaneously hypertensive rats (SHR). VSMCs' proliferation and migration were enhanced by EMs of SHR via the uptake of EMs in VSMCs, but not by EMs of WKY. Proteomics analysis showed that increased osteopontin (OPN) content may be responsible for the roles of EMs of SHR, which was confirmed by the fact that EMs of SHR pretreated with OPN knockdown lost their roles in promoting VSMCs proliferation and migration. OPN successively promoted the phosphorylation of FAK, PI3K and AKT via acting on integrin αVβ3. Inhibition of integrin αVβ3, FAK, PI3K or AKT almost abolished the effects of EMs of SHR on VSMCs proliferation and migration. Knockdown of OPN in the carotid artery attenuated local vascular remodelling in SHR. Repetitive intravenous injection of EMs of SHR increased blood pressure and promoted vascular remodelling in WKY and SHR. We conclude that EMs from VAFs of SHR promote VSMCs proliferation, migration and vascular remodelling via transferring OPN and subsequently activating integrin αVβ3/FAK/PI3K/AKT signalling pathway.

## 1 Introduction

Cells communicate with each other by releasing some signal molecules including proteins, nucleic acids and lipids, which can be packaged by cells in the form of extracellular vesicles (EVs) (Welsh, Goberdhan, O'Driscoll, Théry, et al. 2024) and two non‐vesicular extracellular nanoparticles (NVEPs), exomeres (EMs) and supermeres (SMs) (Jeppesen et al. 2023). EMs and SMs were first reported in 2018 and 2021, respectively. The protocol of isolation and identification of EMs and SMs have been established (Zhang et al. 2023). Unlike small EVs (sEVs) with lipid bilayer membranes and larger sizes (30–100 nm) (Welsh, Goberdhan, O'Driscoll, Buzas, et al. 2024), EMs (28–50 nm) and SMs (22–32 nm) are small and lack membranes (Jeppesen et al. 2023). Several proteins and nucleic acids in sEVs are found in EMs and SMs (Jeppesen et al. 2023). SMs are rich in cargo related to cancers, cardiovascular diseases and Alzheimer's disease, and most extracellular RNAs are associated with SMs rather than sEVs and EMs (Zhang et al. 2021). Recently, it has been reported that EMs and SMs are released from some cells, and the cargo in them is related to several key biological functions (Chitti et al. 2024).

Vascular smooth muscle cells (VSMCs) are the dominant cellular component in the media of arteries and are essential for maintaining the vascular morphology, structure and function (Cao et al. 2022). Enhanced VSMCs proliferation and migration contribute to maladaptive vascular remodelling in several vascular diseases, including hypertension (Totoń‐Żurańska et al. 2024). Vascular remodelling in hypertension refers to the narrowing and thickening of arteries and is involved in a dynamic interplay among various cells and molecular signalling (Zeng and Yang 2024). The maladaptive vascular remodelling is a hallmark of hypertension, and may impair organ flow reserve in the progressive deterioration of hypertension, and is closely associated with hypertension‐related organ damage and cardiovascular events (Rizzoni et al. 2023).

Vascular adventitia is a biological processing centre for the integration of various information, and the production and release of several crucial regulatory factors that regulate vasculature and vascular function (Stenmark et al. 2013). Vascular adventitial fibroblasts (VAFs) are the most abundant cells in the adventitia, and have important partnerships with the VSMCs in tunica media in phenotype transformation, proliferation, migration, apoptosis and contraction as well as vascular remodelling (Sartore et al. 2001). Sympathetic nerves primarily innervate the adventitia of artery (Hirst and Edwards 1989), and sympathetic denervation caused by superior cervical ganglionectomy attenuates vascular remodelling of local arteries innervated by superior cervical ganglion in spontaneously hypertensive rats (SHR) (Wang et al. 2025). Previous studies in our lab have shown that sEVs from VAFs of SHR promote vascular remodelling (Ren et al. 2020; Ye et al. 2022), and norepinephrine, a neurotransmitter of sympathetic nerve, promotes sEVs release from VAFs, and then induces VSMCs proliferation (Ye et al. 2022). However, the role of VAFs in regulating VSMCs is much more complex than that of sEVs. Whether EMs and SMs are involved in VSMCs proliferation, migration and vascular remodelling is unknown. After separating the sEVs from the medium of VAFs from normotensive Wistar‐Kyoto rats (WKY) and SHR, we further isolated EMs and SMs from the supernatant, and found the EMs of SHR (S‐EMs) had an important role in vascular remodelling in SHR. This study is designed to determine the roles and mechanisms of VAFs‐derived EMs in VSMCs proliferation and migration, and vascular remodelling in WKY and SHR.

## 2 Results

### 2.1 Characteristic of VAFs‐Derived sEVs, EMs and SMs

**FIGURE 1:**
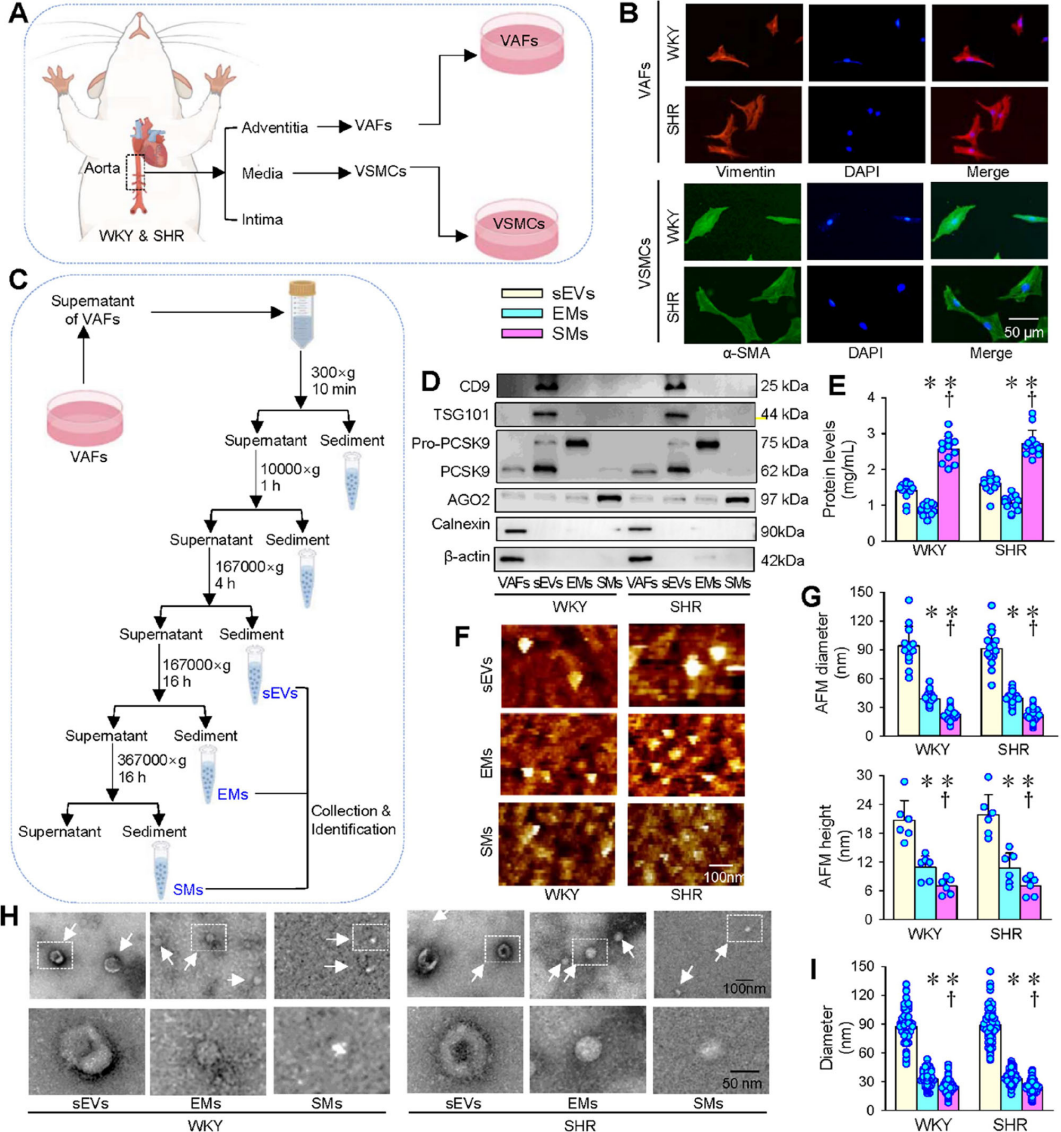
Characteristics of extracellular nanoparticles derived from vascular adventitial fibroblasts of WKY and SHR. (A) A schematic diagram showing the isolation of vascular adventitial fibroblasts (VAFs) and vascular smooth muscle cells (VSMCs) from rat thoracic aorta. (B) Immunofluorescence staining for vimentin (red) in VAFs and α‐SMA (green) in VSMCs. The unclei were stained with DAPI (blue). (C) A schematic diagram showing the isolation procedures of small extracellular vesicles (sEVs), exomeres (EMs) and supermeres (SMs) derived from VAFs. (D) Representative immunoblots of protein markers of sEVs, EMs and SMs. (E) Protein levels in the nanoparticles of 12 batches of isolation from six independent samples of both WKY and SHR. (F) Representative topographic images of fluid‐phase atomic force microscope (AFM) showing the nanoparticles. (G) AFM diameter and AFM height of the nanoparticles obtained from AFM images from six independent samples of both WKY and SHR. (H) Representative images of transmission electron microscope (TEM) showing the nanoparticles. (I) Nanoparticle diameter according to TEM images from six independent samples of both WKY and SHR. Values are mean ± SD. *p < 0.05 versus sEVs; †*p* < 0.05 versus EMs. Two‐way ANOVA followed by Bonferroni post hoc test.

VAFs and VSMCs were isolated from the aorta of WKY and SHR (Figure 1A), and were identified by the VAFs protein marker vimentin and the VSMCs protein marker α‐SMA (Figure 1B). sEVs, EMs and SMs were fractionated from the medium of VAFs with ultracentrifugation (Figure 1C). Typical sEVs‐associated protein markers, tetraspanin CD9 and tumour susceptibility gene 101 (TSG101), were found in sEVs, but not in EMs and SMs. Although proprotein convertase subtilisin/kexin type 9 (PCSK9) appeared in sEVs rather than EMs or SMs, the precursor of PCSK9 (pro‐PCSK9) was abundant in EMs, trace in sEVs, and absent in SMs. Argonaute 2 (AGO2) was abundant in SMs, but trace in sEVs and EMs. Based on these protein markers, we can effectively distinguish sEVs, EMs and SMs from VAFs. Calnexin and β‐actin were used as controls, which only existed in VAFs homogenate, but not in sEVs, EMs and SMs (Figure 1D). Total protein level was highest in SMs, followed by sEVs, and lowest in EMs. The protein level in the EMs of SHR (S‐EMs) was higher than EMs of WKY (W‐EMs) (Figure 1E). Fluid‐phase Atomic force microscopy (AFM) images showed the morphological structure of sEVs, EMs and SMs from VAFs of WKY and SHR (Figure 1F). Based on AFM analyses, both diameter and height were sEVs, EMs and SMs in descending order (Figure 1G). Transmission electron microscope (TEM) images showed that sEVs had vesicles with the largest size, and EMs and SMs were smaller in size in sequence without a membrane (Figure 1H). The TEM image analyses showed that there was no significant difference in sEVs, EMs and SMs diameter between WKY and SHR (Figure 1I).

### 2.2 Effects of EMs and SMs on VSMCs Proliferation and Migration

**FIGURE 2:**
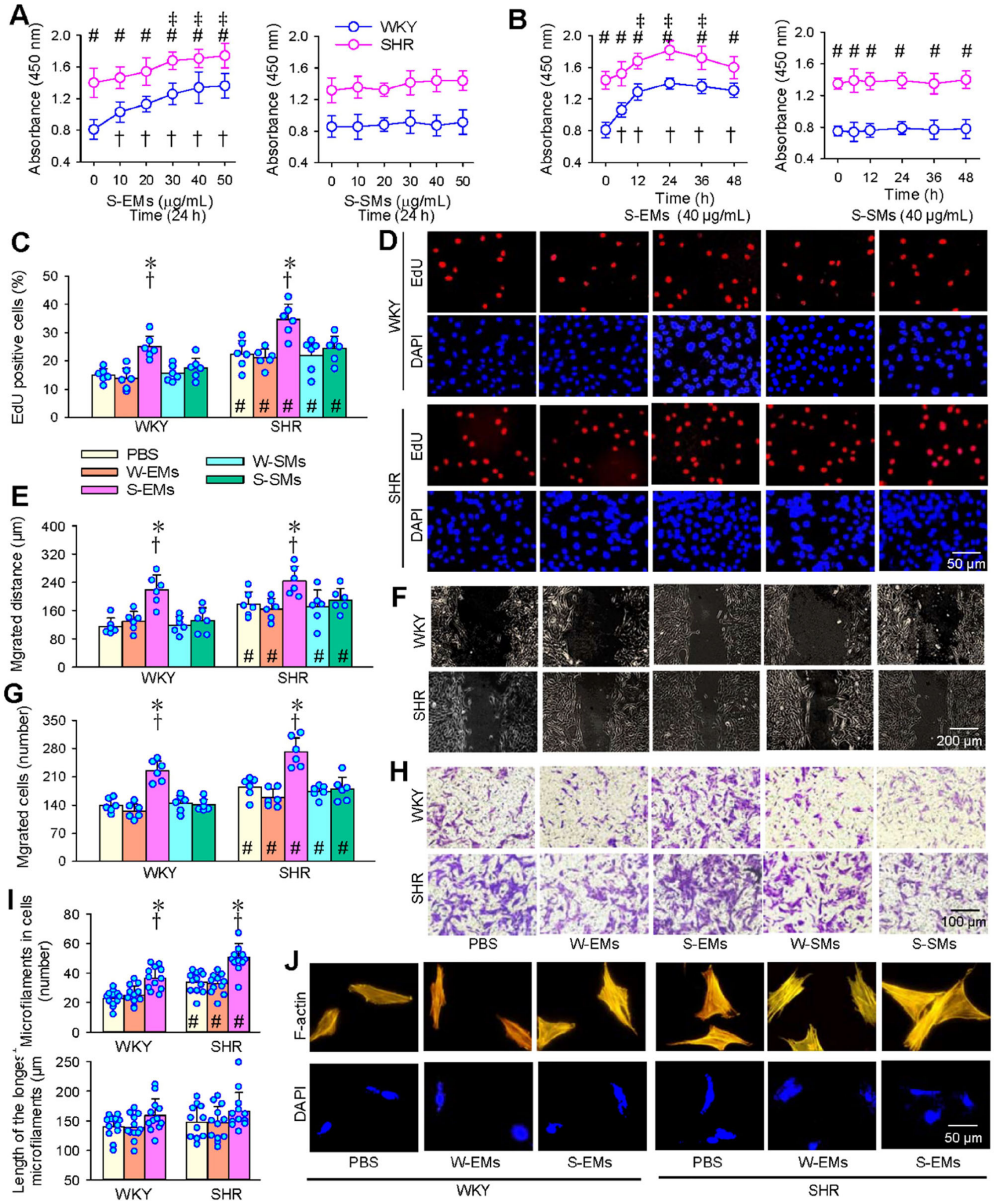
Effects of EMs and SMs derived from VAFs of WKY and SHR on the VSMCs proliferation and migration of WKY and SHR. (A) Dose‐effects of S‐EMs and S‐SMs on VSMCs proliferation evaluated by CCK‐8 kits. (B) Time‐effects of S‐EMs and S‐SMs on VSMCs proliferation evaluated by CCK‐8 kits. (C and D) Effects of EMs and SMs derived from WKY and SHR on VSMC proliferation of WKY and SHR. The VSMCs proliferation was evaluated with EdU‐positive cells (red). The nuclei were stained with DAPI (blue). (E and F) Effects of EMs and SMs derived from WKY and SHR on VSMCs migration evaluated with wound healing assay. (G and H) Effects of EMs and SMs derived from WKY and SHR on VSMCs migration evaluated with Boyden chamber assay. (I and J) Number of microfilaments and length of the longest microfilaments according to F‐actin immunofluorescence analysis. Measurements were made 24 h after transferring EMs or SMs (40 µg/mL) to the media VSMCs of WKY and SHR. Values are mean ± SD. *p < 0.05 versus PBS; †*p* < 0.05 versus W‐EMs; ‡*p* < 0.05 versus 0 µg/mL; #*p* < 0.05 versus WKY. *n* = 12 in (I), and *n* = 6 in others. Repeated measure ANOVA (A, B) and two‐way ANOVA (C, E, G, I) followed by the Bonferroni test.

S‐EMs promoted VSMCs proliferation of both WKY and SHR in a concentration‐dependent manner, determined with CCK‐8 kits, almost reaching its maximal effects at the concentration of 40 µg/mL, and lasting at least 48 h. This concentration was used for the experiments for administration of EMs or SMs. However, SMs from SHR had no significant effects on VSMCs proliferation of both WKY and SHR (Figure 2A,B). The roles of S‐EMs in promoting VSMCs proliferation were further confirmed with EdU Cell Proliferation Assay. W‐EMs or SMs of WKY and SHR had no significant effects on VSMCs proliferation (Figure 2C,D). Similarly, S‐EMs promoted VSMCs migration evaluated with wound healing assay (Figure 2E,F) and Boyden chamber assay (Figure 2G,H). The VSMCs migration was not affected by W‐EMs, or SMs of WKY and SHR. It is known that cell migration is driven by F‐actin polymerisation at the front (Bisaria et al. 2020), and the dynamic regulation of F‐actin cytoskeleton is crucial for several cellular processes, including migration, adhesion and division (Stricker et al. 2010). S‐EMs increased the number of microfilament but had no significant on the length of longest microfilament in the VSMCs of WKY and SHR (Figure 2I,J). The findings confirmed the roles of S‐EMs in promoting VSMCs migration, and S‐EMs may also involve other cellular processes.

### 2.3 Uptake of EMs by VSMCs

**FIGURE 3:**
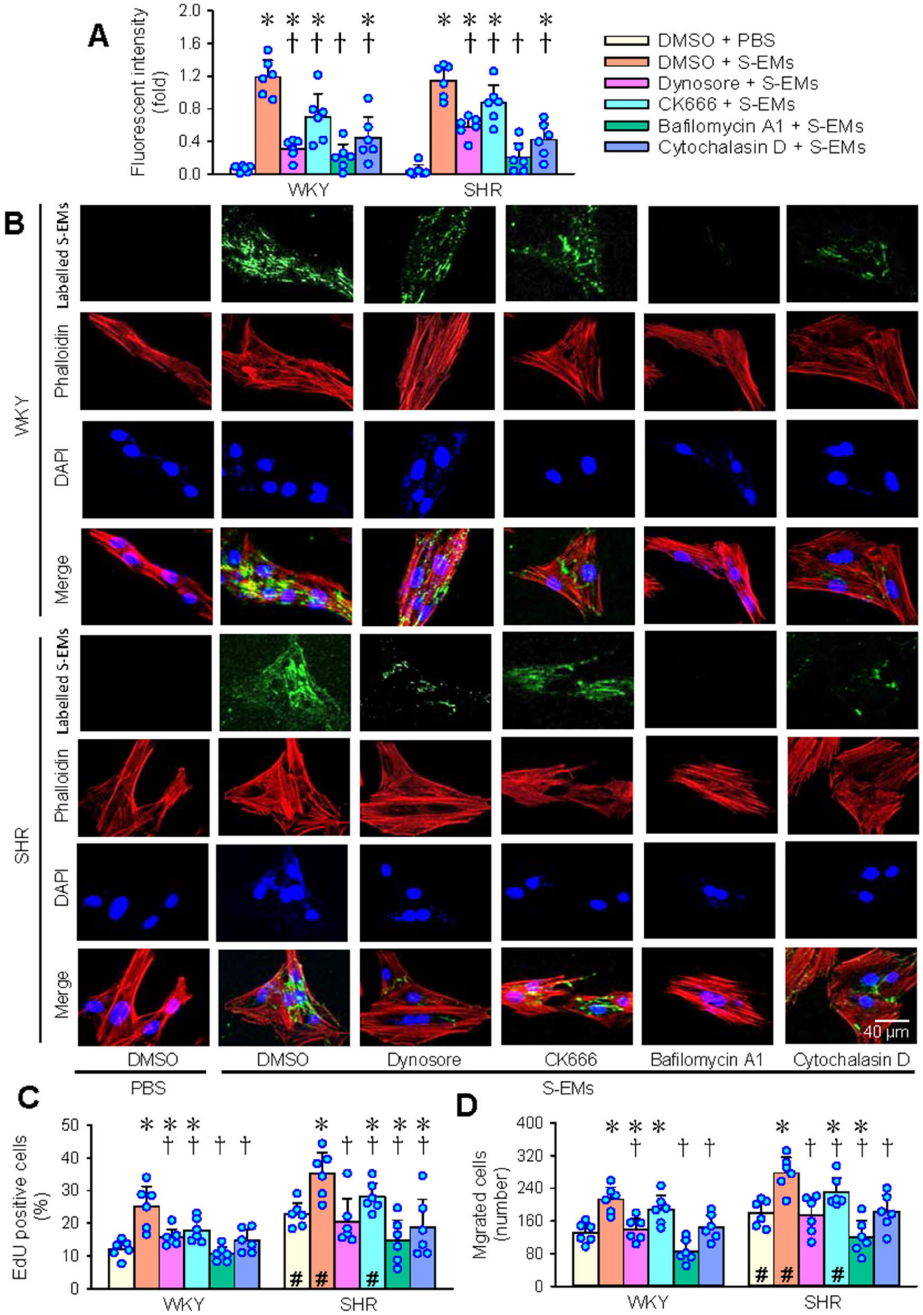
Uptake of S‐EMs in VSMCs of WKY and SHR. (A) Relative fluorescent intensity of labelled S‐EMs in VSMCs. The S‐EMs were labelled with Elab Fluor‐647 NHS ester. The cells were pre‐incubated with 1% DMSO (vehicle), dynasore (20 µM), CK666 (25 µM), bafilomycin A1 (100 nM) or cytochalasin D (5 µM) for 30 min, and then incubated with the labelled S‐EMs for 2 h. (B) Representative images showing the labelled the labelled S‐EMs fluorescence (green) in the VSMCs. Immunofluorescence staining for Phalloidin showing the cytoskeleton (red). Nuclei were stained with DAPI (blue). (C) VSMCs proliferation was evaluated with EdU‐positive cells. (D) VSMCs migration was evaluated with the Boyden chamber assay. Values are mean ± SD. *p < 0.05 versus DMSO + PBS; †*p* < 0.05 versus DMSO + S‐EMs; #*p* < 0.5 versus WKY. *n* = 6. Two‐way ANOVA followed by Bonferroni test.

To examine the uptake of S‐EMs by VSMCs, the S‐EMs were labelled with a fluorescent dye Elab Fluor@647. VSMCs were incubated with the labelled S‐EMs for 2 h to observe the fluorescence in the VSMCs, and the effects of several inhibitors related to different cellular‐uptake mechanisms on the uptake of S‐EMs, proliferation and migration in VSMCs of WKY and SHR were investigated. Dynasore is an endocytosis inhibitor that relies on dynamin (Macia et al. 2006). CK‐666 is an actin‐related protein 2/3 (Arp2/3) complex inhibitor (Sen et al. 2024). Bafilomycin A1 serves as an inhibitor of vacuolar‐type H+‐ATPase (V‐ATPase) (Wang et al. 2021). Cytochalasin D is an inhibitor of the actin‐cofilin interaction by binding to G‐actin (Shoji et al. 2012). Intense intracellular fluorescence was observed in the VSMCs treated with S‐EMs. All four inhibitors reduced intracellular fluorescence, with the strongest effect observed with bafilomycin A1 and the weakest effect observed with CK666 in the VSMCs of both WKY and SHR, suggesting the uptake of S‐EMs by VSMCs through several complex molecular mechanisms (Figure 3A,B). Consistent with the intracellular fluorescence, all four inhibitors attenuated VSMCs proliferation and migration induced by S‐EMs, with the strongest effect in bafilomycin A1‐treated VSMCs and the weakest effect observed with CK666‐treated VSMCs (Figures 3C,D and S1A,B).

### 2.4 Proteomic Analysis of EMs of WKY and SHR

**FIGURE 4:**
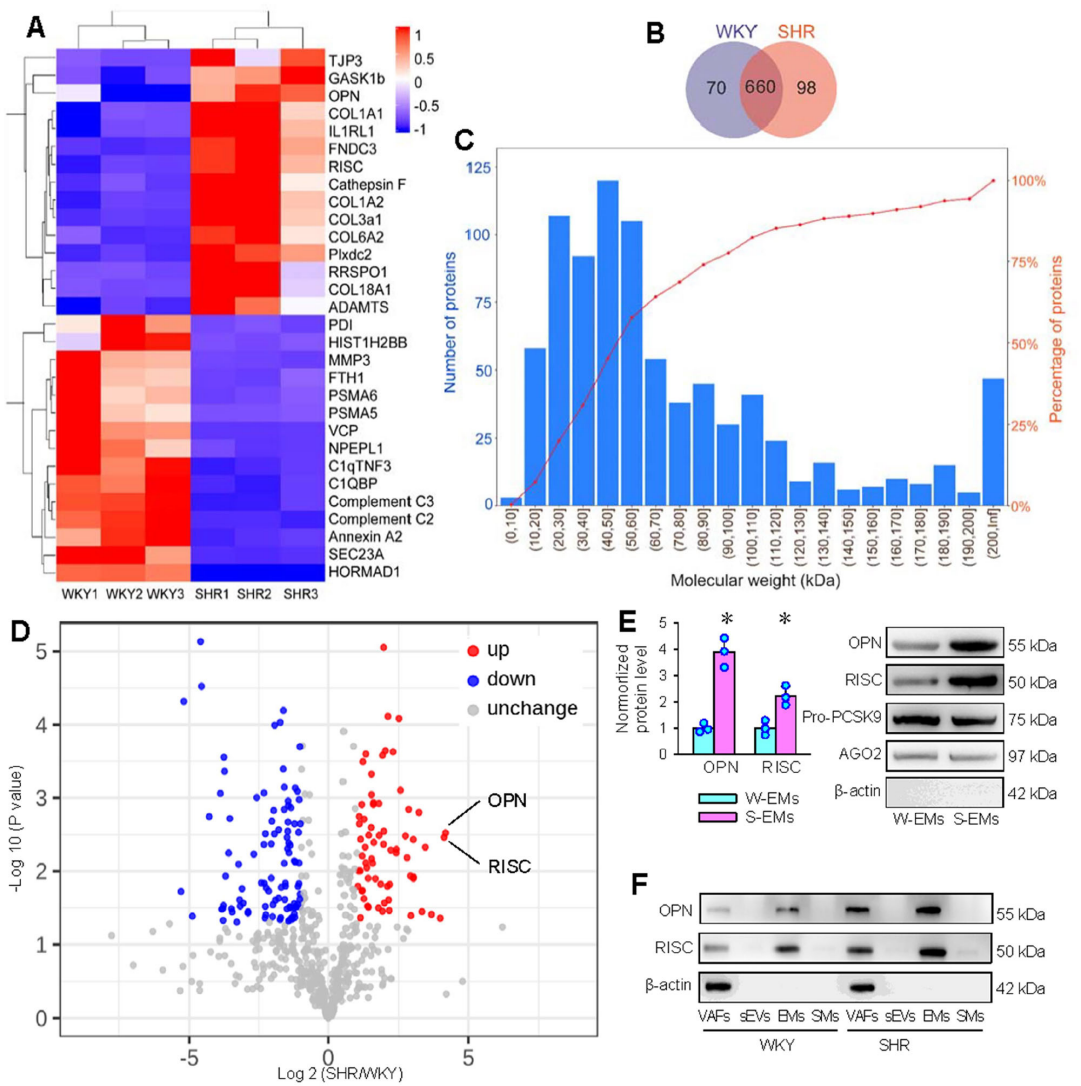
Proteomics analysis of W‐EMs and S‐EMs. (A) Cluster heatmap of the proteins in W‐EMs and S‐EMs. (B) Venn diagram showing the difference of protein in W‐EMs and S‐EMs. (C) Molecular weight distribution of proteins or peptides in the EMs from VAFs. (D) Volcanic map showing the expression of W‐EMs and S‐EMs differential proteins. The horizontal coordinate represents the fold change, and the vertical coordinate represents the significance of the amplitude of change (*p* value). (E) Western blot analysis of OPN and RISC protein levels in EMs of WKY and SHR. Pro‐PCSK9 and AGO2 were used as the loading/normalisation controls (positive controls) and β‐actin was used as a negative control. (F) Representative Western blot images showing OPN and RISC protein in VAFs, sEVs, Ems and SMs. Values are mean ± SD. *p < 0.05 versus W‐EMs. *n* = 3. Unpaired Student's *t*‐test.

Proteomics analysis was performed to reveal the protein difference in the EMs between WKY and SHR. The cluster heatmap displayed 30 proteins with the largest differences, including 15 upregulated proteins and 15 downregulated proteins. These significant differential proteins were shown on the right side of the cluster heatmap (Figure 4A). Venn diagram showed the number of difference proteins in EMs of WKY and SHR (Figure 4B). Volcanic map showed the difference of individual protein contents in the EMs between WKY and SHR. Molecular weight distribution analyses showed that about 80% of the protein molecules in the EMs had a molecular weight between 10 and 110 kDa, of which approximately 52% had a molecular weight between 20 and 60 kDa (Figure 4C). Volcanic map showed the differential proteins in the EMs between WKY and SHR (Figure 4D).

Among the proteins with significant differences displayed in Cluster heatmap (Figure 4A) and Volcanic map (Figure 4D), osteopontin (OPN) and serine carboxypeptidase 1 (RISC or Scpep1) caught our attention because previous studies showed that OPN and RISC were closely associated with cell proliferation and migration (Gadeau et al. 1993; Lee et al. 2009; Vay et al. 2021). Western blot analyses showed that both OPN and RISC contents in the EMs of SHR were higher than that of WKY, in which Pro‐PCSK9 and AGO2 were used as positive controls, and β‐actin was used as a negative control (Figure 4E). Furthermore, either OPN or RISC uniquely existed in EMs, but not in sEVs or SMs (Figure 4F).

### 2.5 Effects of EMs From OPN Knockdown or RISC Knockdown VAFs on VSMCs Proliferation and Migration

**FIGURE 5:**
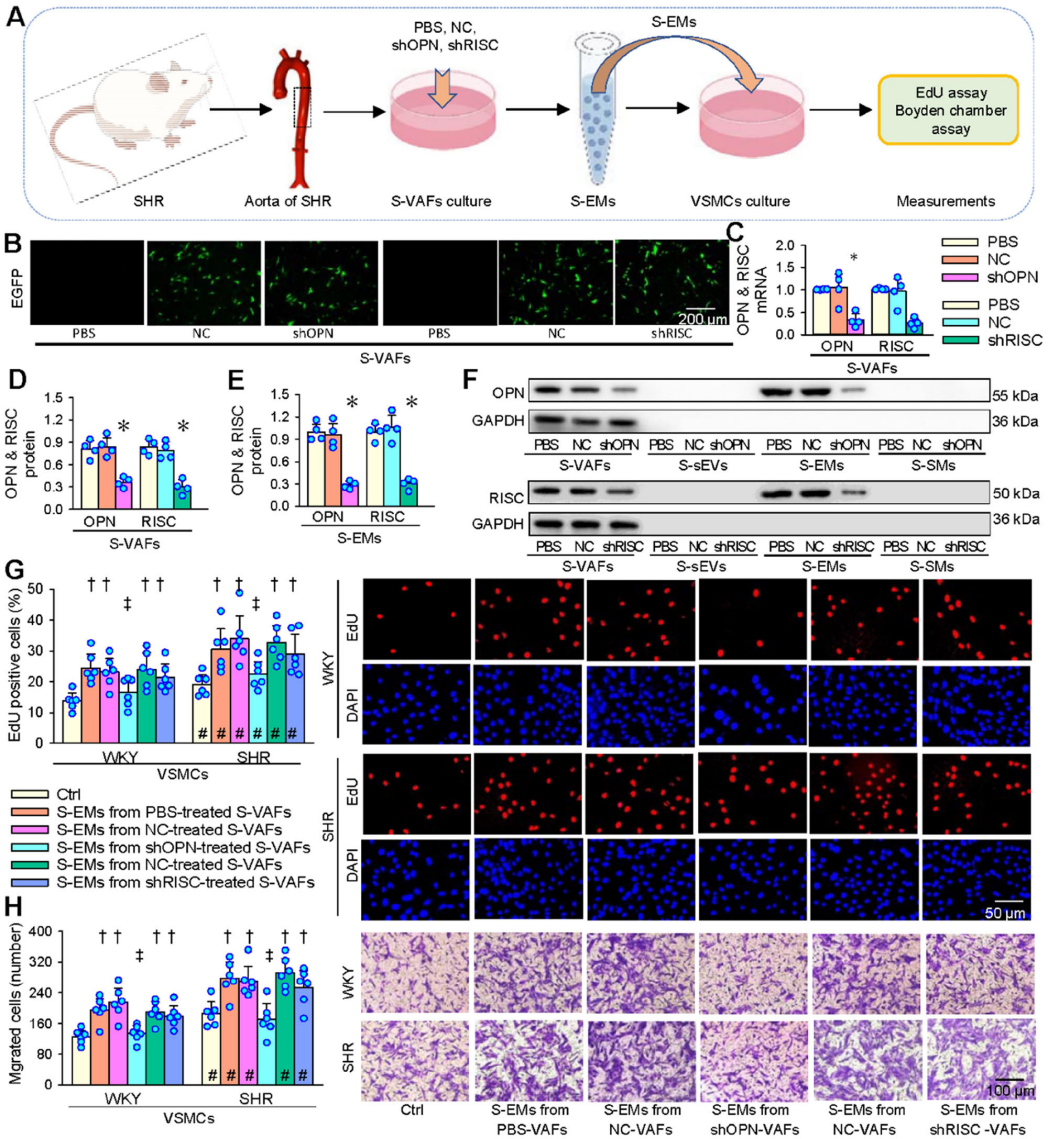
Effects of S‐EMs derived from the S‐VAFs subjected to OPN or RISC knockdown on VSMC proliferation and migration of WKY and SHR. VAFs from SHR were infected with OPN shRNA encoded‐lentiviral vector (shOPN, MOI = 10) or RISC shRNA encoded‐lentiviral vector (shRISC, MOI = 80) for knockdown of OPN or RISC. PBS and negative control shRNA encoded‐lentiviral vector (NC) were used as controls. (A) Schematic diagram showing the experimental protocol. (B) EGFP expressing cells 72 h after the transfection. (C) OPN and RISC mRNA levels in VAFs of SHR 5 days after the transfection. (D) OPN and RISC protein levels in VAFs of SHR 5 days after the transfection. (E) OPN and RISC protein contents in the S‐EMs derived from S‐VAFs pretreated with PBS, NC, shOPN or shRISC. (F) Representative Western blot images. (G and H) Effects of EMs from OPN KD‐ or RISC KD‐treated S‐VAFs on VSMCs proliferation (G) and migration (H) of WKY and SHR. The cell proliferation and migration were evaluated 24 h after application of the EMs by the percentage of EdU‐positive cells and Boyden chamber assay, respectively. Values are mean ± SD. *p < 0.05 versus PBS or NC; †*p* < 0.05 versus Ctrl; ‡*p* < 0.05 versus S‐EMs from PBS‐ or NC‐treated VAFs. #*p* < 0.05 versus WKY. *n* = 4 in (C–E); *n* = 6 in (G and H). One‐way ANOVA (C–E), and two‐way ANOVA followed by Bonferroni test (G, H).

To determine whether OPN or RISC in the S‐EMs contributes to VSMCs proliferation and migration, OPN‐ or RISC‐shRNA encoded‐lentiviral vectors was used to construct stable transmutation strains with OPN knockdown or RISC knockdown in VAFs of SHR. A schematic diagram shows the experimental protocol for determining the effects of EMs from these pretreated VAFs on VSMCs proliferation and migration (Figure 5A). The effectiveness of lentiviral transfection was confirmed by the fluorescence of EGFP expression in VAFs (Figure 5B), reduced OPN and RISC mRNA and protein expressions in VAFs, and diminished OPN and RISC protein contents in S‐EMs (Figure 5C–F). No OPN or RISC protein existed in sEVs or SMs (Figure 5F). S‐EMs from OPN knockdown‐treated VAFs of SHR almost lost their roles in promoting VSMCs proliferation and migration measured 24 h after application of the S‐EMs, while S‐EMs from RISC knockdown‐treated VAFs of SHR still remained their roles in promoting VSMCs proliferation and migration of WKY and SHR (Figure 5G,H), indicating that S‐EMs promote VSMCs proliferation and migration by transferring OPN rather than RISC. OPN antibody in VSMCs of WKY and SHR inhibited the roles of S‐EMs in promoting VSMCs proliferation and migration (Figure S2A,B). OPN knockdown in VAFs of SHR had no significant effects on total protein, morphology under transmission electron microscopy, and size of sEVs, EMs and SMs (Figure S3A–C), suggesting that OPN knockdown did not affect the release and basic characteristic of S‐EMs, but only reduced the OPN content in the S‐EMs.

### 2.6 Integrin αVβ3 Mediates the Roles of OPN in the EMs in Promoting VSMCs Proliferation and Migration

**FIGURE 6:**
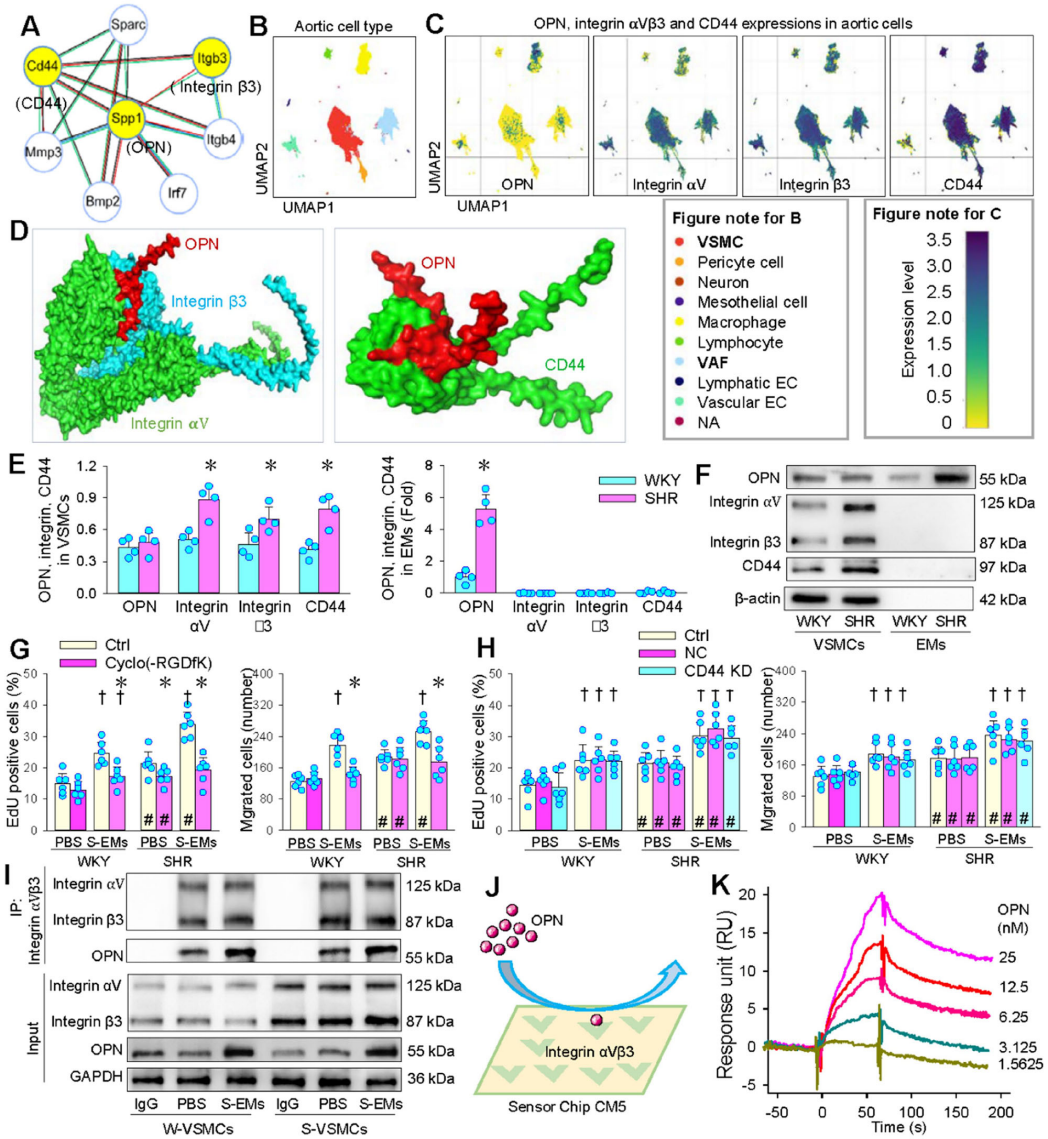
Interaction of OPN with integrin αVβ3 and CD44, and the roles of integrin αVβ3 and CD44 in S‐EMs‐induced VSMCs proliferation and migration of WKY and SHR. (A) Protein interactions of Spp1 predicted with STRING database. SPP1, Cd44 and Itgb3 are the protein coding gene of OPN, CD44 and integrin β3, respectively. (B and C) UMAP plot showing the classification of human aortic cells (B), and their expressions of OPN, integrin αV, integrin β3 and CD44 (C) according to single‐cell sequencing data of human aortic cells from three individuals according to the Single Cell database. VSMC, vascular smooth muscle cell; EC, endothelial cell; VAF, vascular adventitial fibroblast. (D) Molecular docking of OPN (red) and integrin αV (green) and β3 (blue), and molecular docking of OPN (red) and CD44 (green). (E) Levels of OPN, integrin αVβ3, and CD44 in VSMCs and EMs of WKY and SHR. (F) Representative Western blot images. (G) Effects of integrin αVβ3 inhibitor Cyclo (‐RGDfK) on S‐EMs‐induced VSMCs proliferation and migration of WKY and SHR. The integrin αVβ3 inhibitor Cyclo (‐RGDfK) (10 µM) was administered 2 h before S‐EMs (40 µg/mL). (H) Effects of CD44 knockdown (KD) on the S‐EMs‐induced VSMCs proliferation and migration of WKY and SHR. The si‐CD44 (50 nM) was administered 48 h before S‐EMs (40 µg/mL). VSMCs proliferation and migration were evaluated by the percentage of EdU‐positive cells and Boyden chamber assay 24 h after application of the S‐EMs. (I) Co‐immunoprecipitation (Co‐IP) and western blot images showing the binding of OPN with integrin αVβ3 in VSMCs. (J) Schematic of a gold surface with integrin αVβ3 protein immobilised on a sensor chip CM5 in surface plasmon resonance (SPR) assay. (K) Sensorgrams depicting interaction of OPN and integrin αVβ3. Values are mean ± SD. *p < 0.05 versus Ctrl; †*p* < 0.05 versus PBS; #*p* < 0.05 versus WKY. *n* = 4 in (E); *n* = 6 in others. Two‐way ANOVA followed by Bonferroni test (G–I). Unpaired Student's *t*‐test (E).

STRING database (https://cn.string‐db.org/) was used to construct the protein‐protein interaction (PPI) of OPN. Secreted phosphoprotein 1 (Spp1, coding gene of OPN) is closely related to secreted protein acidic and rich in cysteine (Sparc), integrin β3 (Itgb3, coding gene of integrin β3), integrin β4 (Itgb4), interferon regulatory factor 7 (Irf7), bone morphogenetic protein 2 (Bmp2) and Cd44 (coding gene of CD44) (Figure 6A). It has been found that the integrin β3 is involved in the role of OPN in promoting VSMCs phenotypic transformation in varicose veins (Jiang et al. 2014), and exosomal OPN from renal tubular cells promoted fibroblast proliferation by binding with CD44 (Chen et al. 2022). Genome‐wide association studies (GWAS) were made in individuals’ thoracic aorta data from the Single Cell database (https://singlecell.broadinstitute.org/single_cell) to analyse the cell types of the thoracic aorta, and the expressions of OPN, integrin αVβ3 and CD44. VSMCs and VAFs were the most numerous cells (Figure 6B). Mild to moderate expression of OPN, and high expression of integrin αVβ3 and CD44 were found in the VSMCs and VAFs (Figure 6C). Protein‐protein docking showed that OPN bound to both integrin αVβ3 and CD44 (Figure 6D). The predicted binding sites and intermolecular energy landscape in protein interaction were shown in the online supplementary data (Figures S4 and S5). There was no significant difference in OPN protein levels in VSMCs between WKY and SHR, but integrin αV, integrin β3 and CD44 proteins was significantly upregulated in VSMCs of SHR compared with those of WKY. OPN content in the EMs of SHR was about five times that of WKY. No integrin αV, integrin β3 and CD44 proteins were found in sEVs and SMs of WKY and SHR (Figure 6E,F). Cyclo(‐RGDfK) is a selective integrin αVβ3 inhibitor (Okada et al. 2021). Pretreatment with Cyclo(‐RGDfK) prevented the S‐EMs induced proliferation and migration of WKY and SHR 24 h after application of the S‐EMs (Figure 6G). CD44‐siRNA (siCD44) effectively reduced CD44 mRNA and protein expressions in VSMCs of WKY and SHR (Figure S6), but had no significant effects on the S‐EMs induced VSMCs proliferation and migration of WKY and SHR 24 h after application of the S‐EMs (Figure 6H). These results indicate that the roles of S‐EMs in promoting VSMCs proliferation and migration were mediated by the action of OPN on integrin αVβ3. Thus, co‐immunoprecipitation (Co‐IP) assay and SPR were performed to provide further evidence for the OPN‐integrin αVβ3 interaction. Co‐IP assay showed the combination of OPN with integrin αVβ3 (Figure 6I). Surface plasmon resonance (SPR) assay revealed the binding of OPN to integrin αVβ3 protein fixed on the surface of the sensor chip CM5 in a concentration‐dependent manner (Figure 6J,K).

### 2.7 Integrin αVβ3/FAK/PI3K/AKT Signalling Pathway Mediates Effects of EMs of SHR

**FIGURE 7:**
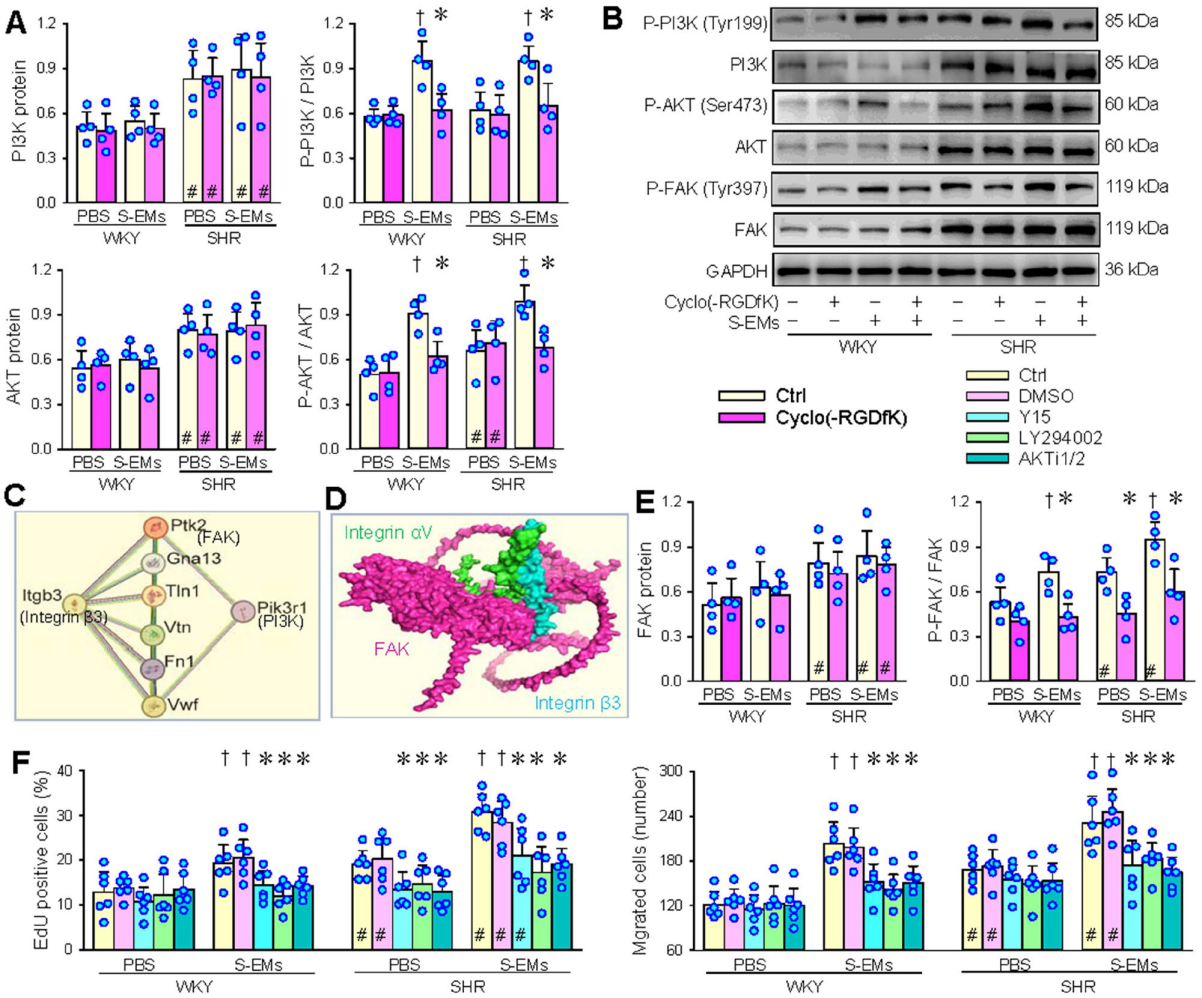
Integrin αVβ3‐FAK‐PI3K‐AKT signalling mediates the roles of S‐EMs in promoting VSMCs proliferation and migration of WKY and SHR. (A) Effects of integrin αVβ3 inhibitor Cyclo(‐RGDfK) (10 µM) on S‐EMs‐induced PI3K (p85α) and AKT phosphorylation in VSMCs. (B) Representative Western blot images. (C) Protein interactions of integrin β3 with PI3K according to STRING database. Itgb3, Pik3r1 and PIk2 are the protein coding gene of integrin β3, PI3K and FAK, respectively. (D) Molecular docking of integrin αV (green) and β3 (blue), and FAK (purple). (E) Effects of integrin αVβ3 inhibitor Cyclo(‐RGDfK) (10 µM) on S‐EMs‐induced FAK phosphorylation in VSMCs. (F) Effects of FAK inhibitor Y15 (2.5 µM), PI3K inhibitor LY294002 (5 µM) or AKT inhibitor AKTi1/2 (0.5 µM) on S‐EMs‐induced VSMC proliferation and migration. S‐EMs (40 µg/mL) was administered 2 h after application of the inhibitors. VSMCs proliferation and migration were evaluated by the percentage of EdU‐positive cells and Boyden chamber assay. Values are mean ± SD. *p < 0.05 versus Ctrl or DMSO; †*p* < 0.05 versus PBS; #*p* < 0.05 versus WKY. *n* = 4 in (A) and (E); *n* = 6 in (F). Two‐way ANOVA followed by Bonferroni test.

Integrin αVβ3/PI3K/AKT pathway impacts various aspects of astrocyte behavior (Pérez‐Núñez et al. 2025), and mediates the effect of milk fat globule epidermal growth factor 8 on hippocampal neurogenesis (Li et al. 2024). The phosphorylation of PI3K and AKT (P‐PI3K and P‐AKT) involves VSMCs proliferation induced by insulin (Isenovic et al. 2009). Compared with WKY, PI3K and AKT protein expressions increased in the VSMCs of SHR, but the P‐PI3K/PI3K and P‐AKT/AKT ratios did not increase due to the similar degree of increase in P‐PI3K and P‐AKT levels. S‐EMs had no significant effects on PI3K and AKT protein levels, but increased the P‐PI3K/PI3K and P‐AKT/AKT ratios in VSMCs of WKY and SHR, which were prevented by integrin αVβ3 inhibitor Cyclo(‐RGDfK) (Figure 7A,B). It has been found that integrin αVβ3 mediates inflammation by activating the FAK‐PI3K‐AKT‐NFkB signalling pathway during porcine deltacoronavirus infection (Jin et al. 2024). PPI map shows that focal adhesion kinase (FAK) encoded by protein tyrosine kinase 2 (PTK2) gene is involved in the interaction between integrin β3 and PI3K (Figure 7C). Protein‐protein docking shows the binding of integrin αVβ3 to FAK (Figure 7D), and the predicted binding sites and intermolecular energy landscape in protein interaction (Figure S7). Similar to PI3K and AKT, S‐EMs had no significant effect on FAK protein expression, but promoted FAK phosphorylation in VSMCs of WKY and SHR, which were prevented by Cyclo(‐RGDfK) (Figure 7E). The roles of S‐EMs in promoting VSMCs proliferation and migration of WKY and SHR were prevented by FAK inhibitor Y15, PI3K inhibitor LY294002 or AKT inhibitor AKTi1/2 (Figure 7F). These results indicate that the effects of S‐EMs on VSMCs proliferation and migration were mediated by integrin αVβ3/FAK/PI3K/AKT signalling pathway.

### 2.8 Effects of OPN Knockdown in Carotid Artery on Local Vascular Remodelling

**FIGURE 8:**
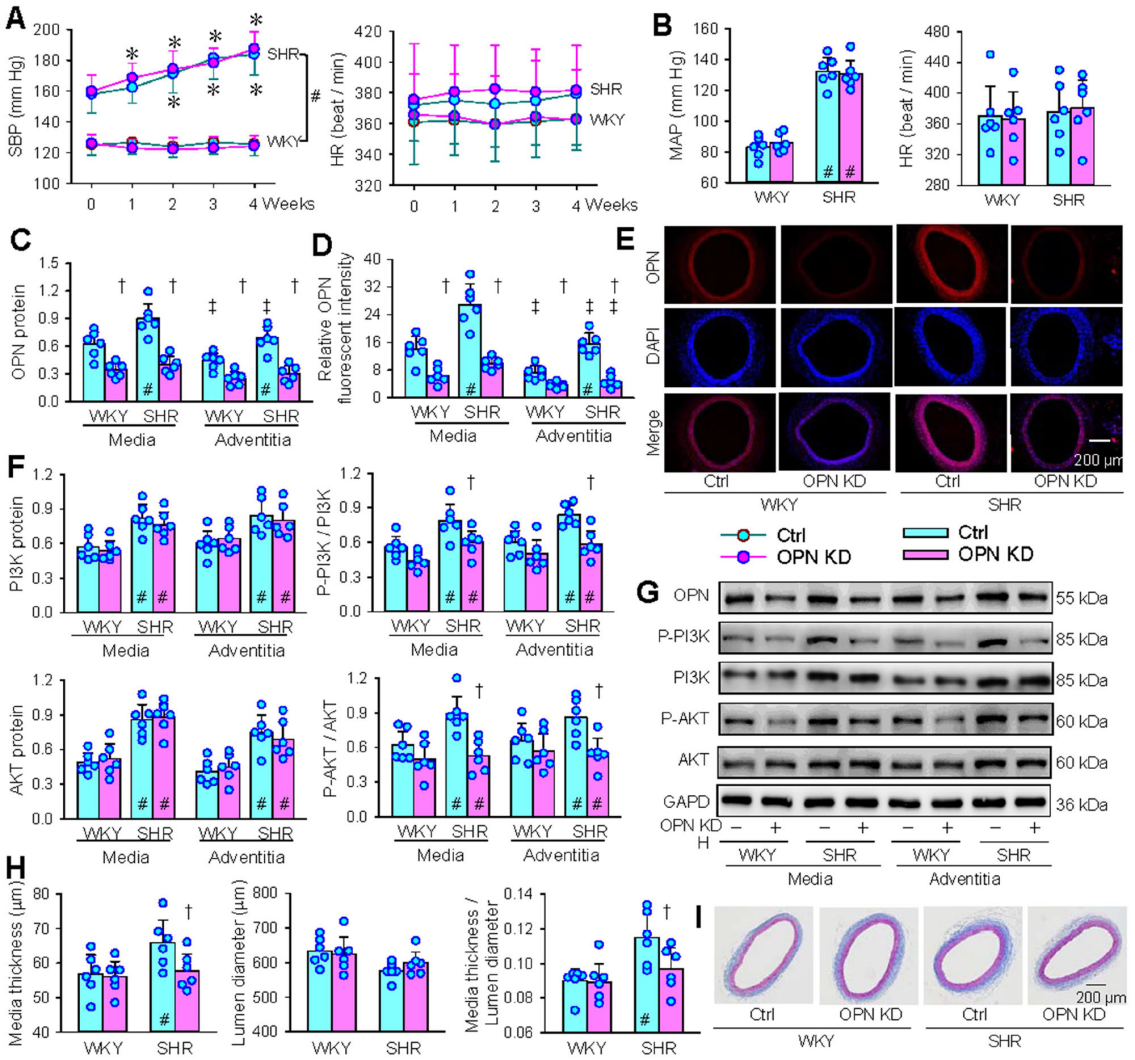
Effects of local knockdown of OPN in carotid artery on blood pressure and vascular remodelling in WKY and SHR. Adeno‐associated‐virus shOPN (AAV‐shOPN, 7 × 10^12^ v.g/mL) was used for OPN knockdown. AAV‐negative control shRNA was used as a control (Ctrl). (A) Systolic blood pressure (SBP) and heart rate (HR) were measured every week in a conscious state. (B) Mean arterial pressure (MAP) and HR were measured under anesthesia 4 weeks after the transfection. (C) OPN protein level (OPN/GAPDH) in tissue lysis of media and adventitia of the carotid artery. (D) Relative OPN fluorescence intensity in adventitia and media of carotid arteries according to OPN immunofluorescence staining. (E) Representative images showing OPN (red) immunofluorescence in carotid arteries. Nuclei were stained with DAPI (blue). (F) Phosphorylation of PI3K and AKT. (G) Representative Western blot images. (H) Media thickness, lumen diameter and the ratio of lumen diameter to media thickness of the carotid artery. (I) Representative images showing the Masson's staining of carotid artery. Values are mean ± SD. *p < 0.05 versus 0 weeks; †*p* < 0.05 versus Ctrl; ‡*p* < 0.05 versus Media; #*p* < 0.05 versus WKY. *n* = 6. Repeated measure ANOVA (A) and two‐way ANOVA (B–E) followed by Bonferroni test.

Local OPN knockdown with AAV‐shOPN in carotid artery was performed in WKY and SHR to examine the role of OPN in vascular remodelling. The OPN knockdown in carotid artery had no significant effect on blood pressure and heart rate in WKY and SHR under both awake state (Figure 8A) and anaesthesia state (Figure 8B). Four weeks after AAV‐shOPN or control treatment, carotid arteries were harvested for measurements. OPN protein in the tissue lysis of media and adventitia of carotid artery was upregulated in SHR compared with WKY, and OPN protein expression in media of WKY and SHR was higher than that in adventitia. The effectiveness of OPN knockdown was confirmed by the downregulation of OPN in the media and adventitia of carotid artery of both strains (Figure 8C). The findings were further confirmed by OPN immunofluorescence analysis (Figure 8D,E). OPN knockdown had no significant effect on PI3K and AKT expressions, but reduced the phosphorylation levels of PI3K and AKT in SHR (Figure 8F,G). Masson's staining showed that the media thickness and the ratio of media thickness to lumen diameter were greater in carotid artery of SHR, which were attenuated by local OPN knockdown (Figure 8H,I). These results suggest vascular OPN plays an important role in vascular remodelling of SHR, being independent of blood pressure.

### 2.9 Effects of EMs on Blood Pressure and EMs Biodistribution

**FIGURE 9:**
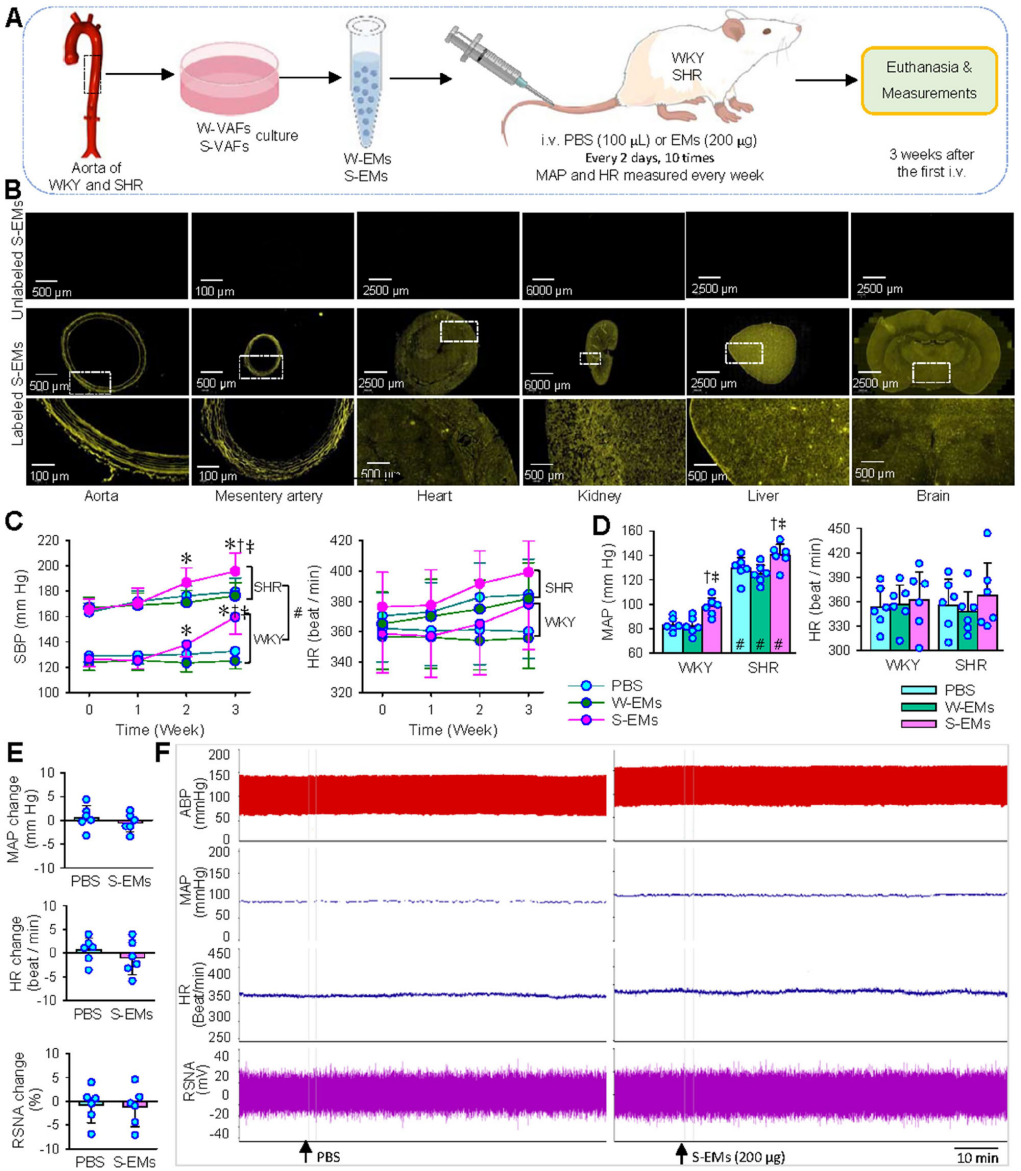
Effects of intravenous injections of VAFs‐derived EMs (200 µg, every 2 days, 10 times) on blood pressure and heart rate in WKY and SHR. (A) Schematic diagram showing the experimental protocol. (B) Representative images showing the S‐EMs distribution in aorta, mesentery artery, heart, kidney, liver, brain of rats 24 h after intravenous injection of Elab Fluor 647 labelled S‐EMs (yellow fluorescence) or unlabelled S‐EMs (without fluorescence). (C) Systolic blood pressure (SBP) and heart rate (HR) measured every week in conscious state. (D) Mean arterial pressure (MAP) and HR measured under anaesthesia 3 weeks after the first injection of PBS, W‐EMs or S‐EMs. (E) Acute effects of single intravenous injection of S‐EMs (200 µg, 200 µL) in WKY on mean arterial pressure (MAP), HR and renal sympathetic nerve activity (RSNA). (F) Representative recordings of arterial blood pressure (ABP), MAP, HR and RSNA changes in WKY after injection of PBS (left panel) and S‐EMs (right panel). Values are mean ± SD. *p < 0.05 versus 0 weeks; †*p* < 0.05 versus PBS; ‡*p* < 0.05 versus W‐EMs; #*p* < 0.05 versus WKY. *n* = 6. Repeated measure ANOVA (C) and two‐way ANOVA (D) followed by Bonferroni test. Unpaired Students’ test (E).

W‐EMs and S‐EMs were intravenously injected into WKY and SHR every 2 days for a total of 10 injections to determine the effects of EMs on blood pressure and vascular remodelling (Figure 9A). The biodistribution of S‐EMs was observed in rats 24 h after intravenous injection of Elab Fluor 647 labelled S‐EMs. The labelled S‐EMs were widely distributed in various tissues and organs. Strong fluorescence was found in arteries, kidneys and liver, which may be related to the abundant blood supply. Moderate fluorescence was found in the brain, including several nuclei involved in regulating sympathetic activity and blood pressure, such as the hypothalamic paraventricular nucleus (PVN). Low fluorescence was found in the heart. Unlabelled S‐EMs was used as a control, and no fluorescence was found in these tissues after intravenous injection of unlabelled S‐EMs (Figure 9B). The S‐EMs significantly increased blood pressure in the 3rd week, but not in the 1st and 2nd weeks in awake WKY and SHR (Figure 9C). Similarly, the S‐EMs increased the blood pressure of anaesthetized WKY and SHR, but did not increase the heart rate at the end of 3rd week (Figure 9D). However, W‐EMs had no significant effects on blood pressure and heart rate in WKY and SHR (Figure 9C,D). It is interesting to understand whether the impact of EMs is related to sympathetic activation. Therefore, the acute effects of a single intravenous injection of S‐EMs (200 µg, 200 µL) on mean arterial pressure (MAP), HR and renal sympathetic nerve activity (RSNA) were examined in WKY. Intravenous injection of S‐EMs had no significant effects on the RSNA, MAP and HR, suggesting that the S‐EMs had no direct effect on sympathetic activation (Figure 9E,F).

### 2.10 Effects of EMs on Blood Pressure and Vascular Remodelling in WKY and SHR

**FIGURE 10:**
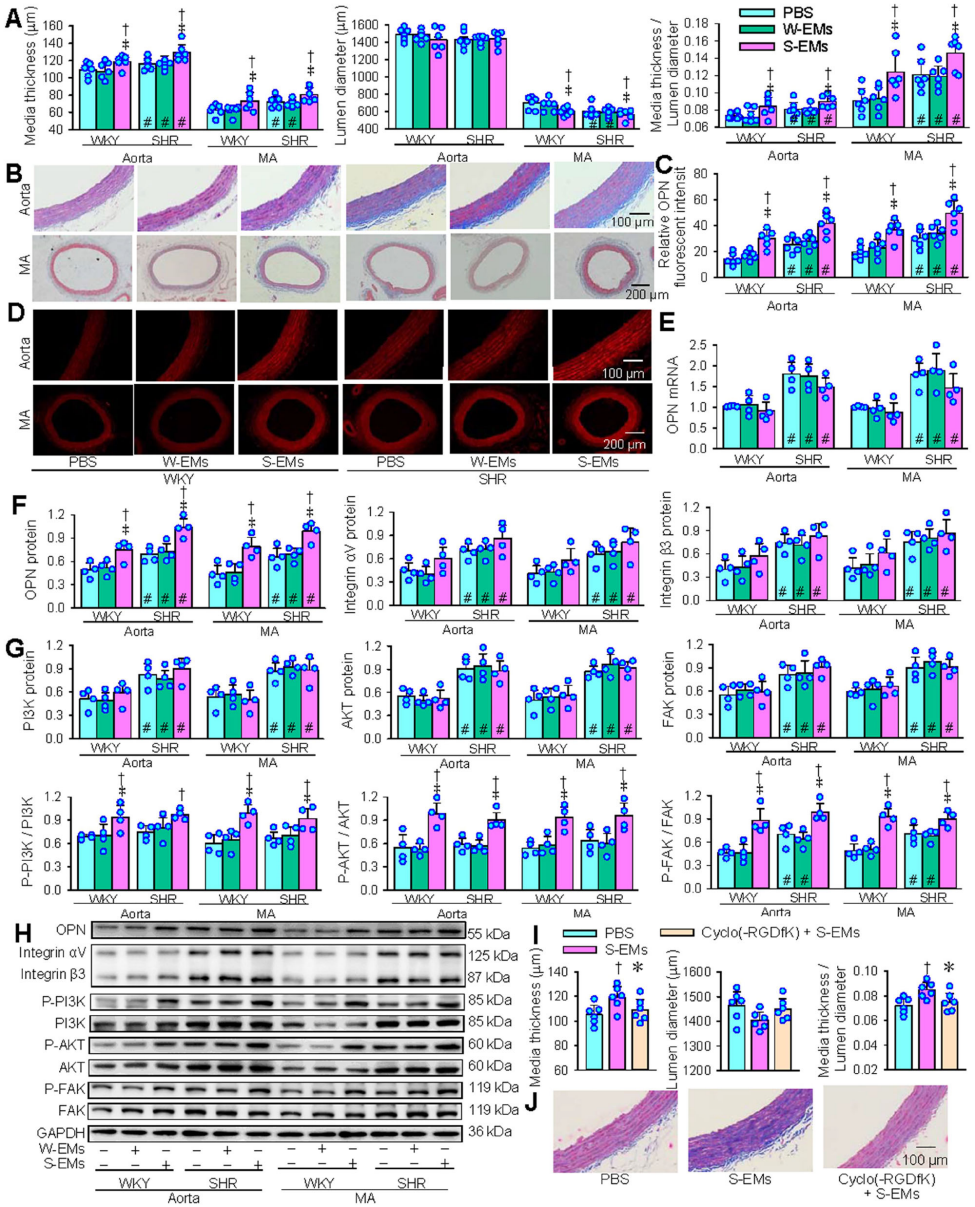
Effects of intravenous injections of VAFs‐derived EMs (200 µg, every 2 days, 10 times) on vascular remodelling and related signalling pathway in aorta and MA of WKY and SHR. (A) Media thickness, lumen diameter and the ratio of lumen diameter to media thickness of the aorta and mesentery artery. (B) Representative images showing the Masson’ staining of aorta and MA. (C) Bar graph showing the relative fluorescent intensity of OPN. (D) Representative images showing OPN immunofluorescence staining. (E) OPN mRNA level; (F) OPN, integrin αV and integrin β3 protein expressions. (G) PI3K, AKT and FAK protein expressions and their phosphorylation levels. (H) Representative Western blot images. (I) Effects of integrin αVβ3 inhibitor Cyclo (‐RGDfK) (10 mg/kg, ip, every 2 days, 10 times) on the roles of S‐EMs (200 µg, iv, every 2 days, 10 times) in promoting vascular remodelling in aorta of WKY. (J) Representative images of the Masson’ staining. Values are mean ± SD. *p < 0.05 versus S‐EMs; †*p* < 0.05 versus PBS; ‡*p* < 0.05 versus W‐EMs; #*p* < 0.05 versus WKY. *n* = 6 in (A), (C) and (I); *n* = 4 in (E–G). Two‐way ANOVA followed by Bonferroni test.

Masson's staining images of aortas showed that S‐EMs increased media thickness and the ratio of media thickness to lumen diameter in both WKY and SHR, but had no significant effects on lumen diameter. The images of mesentery arteries showed that the S‐EMs increased media thickness and the ratio of media thickness to lumen diameter, but reduced lumen diameter in WKY and SHR. However, W‐EMs had no significant effect on vascular remodelling of WKY and SHR (Figure 10A,B). OPN immunofluorescence analysis showed that OPN levels were increased in both the aorta and mesentery artery of the S‐EMs‐treated WKY and SHR (Figure 10C,D). OPN mRNA and protein levels in aorta and mesentery arteries were higher in SHR than WKY, and S‐EMs increased OPN protein levels rather than OPN mRNA levels (Figure 10E,F). Both integrin αV and integrin β3 expressions were upregulated in VSMCs of SHR compared with WKY. However, S‐EMs only induced a tendency in increasing integrin αV and integrin β3 expressions, but these changes did not reach a significant level (Figure 10F). S‐EMs promoted the phosphorylation of PI3K and AKT in aorta and mesentery artery of both strains. However, W‐EMs had no significant effects on these protein expressions and phosphorylation levels of WKY and SHR (Figure 10G,H). To confirm the roles of integrin αVβ3 signalling pathway in mediating the effects of S‐EMs in vivo, the effects of integrin αVβ3 inhibitor Cyclo (‐RGDfK) (10 mg/kg, ip, every 2 day, 10 times) on the roles of S‐EMs (200 µg, iv, every 2 days, 10 times) in promoting vascular remodelling and phosphorylation of PI3K, AKT and FAK were investigated in WKY. Measurements were performed 3 weeks after the first injections. S‐EMs induced vascular remodelling, which were almost abolished by Cyclo (‐RGDfK) (Figure 10I,J). Consistently, S‐EMs induced phosphorylation of PI3K, AKT and FAK were almost abolished by Cyclo (‐RGDfK) (Figure S8A,B). These results indicate that S‐EMs promote vascular remodelling in WKY and SHR, and further support our findings in vitro that the effects of S‐EMs were mediated by integrin αVβ3/FAK/PI3K/AKT signalling pathway.

## 3 Discussion

Vascular remodelling contributes to organ damage and cardiovascular events in hypertension (Rizzoni et al. 2023). VAFs in adventitia are closely related to VSMCs proliferation, migration and vascular remodelling (Sartore et al. 2001). This study shows a novel mechanism of vascular VAFs in modulating the phenotypes of VSMCs and vascular remodelling in hypertension. EMs from VAFs from aorta of SHR promote VSMCs proliferation and migration by transferring OPN to VSMCs and then causing the activation of integrin αVβ3/FAK/PI3K/AKT signalling. The EMs from SHR stimulate vascular remodelling and cause hypertension in normotensive rats, and deteriorate vascular remodelling and hypertension in hypertensive rats. Knockdown of OPN in carotid artery attenuates local vascular remodelling in hypertensive rats. EMs and OPN may be important targets for intervention of vascular remodelling in hypertension.

sEVs‐mediated signalling molecule transfer is one of the local and intercellular communication modes. NVEPs, including EMs and SMs have recently been identified as extracellular nanoparticles contributing to intercellular communications, which lack lipid bilayer membranes and are abundant in extracellular space and body fluids (Zhang et al. 2018, 2021). EMs and SMs are distinct from sEVs in size, morphology, composition, cellular uptake dynamics and tissue distribution (Jeppesen et al. 2023). Previous studies in our lab have found that sEVs from SHR promote vascular remodelling in WKY and SHR. Here, we first isolated and identified EMs and SMs from VAFs of WKY and SHR, and compared them with sEVs. Both AFM and TEM images displayed the typical morphology and size of sEVs, EMs and SMs, with sEVs being large, EMs being medium and SMs being small. SMs had the highest total protein content, followed by sEVs, and EMs had the lowest. In addition to the size and morphology, specific protein markers are important for distinguishing sEVs, EMs and SMs. We tried several possible markers and found some protein markers for distinguishing them. CD9, TSG 101 and PCSK9 (60 kDa) only appeared in sEVs, Pro‐PCSK9 (75 kDa), OPN and RISC merely existed in EMs, and AGO2 was exclusively found in SMs. Furthermore, calnexin and β‐actin existed in whole VAFs lysate, but not in sEVs, EMs and SMs. These findings indicate that these protein markers can effectively distinguish these nanoparticles and suggest the high purity of each kind of nanoparticle obtained in the present study. There was no significant difference between WKY and SHR in the size and total protein level of sEVs, EMs and SMs, suggesting the functional difference between WKY and SHR lies not in the number of nanoparticles, but in some specific component differences, such as OPN and RISC in the EMs of SHR being higher than those in WKY.

EMs of SHR stimulated VSMCs proliferation and migration of WKY and SHR, but EMs of WKY, SMs of WKY and SHR had no significant effects. Thus, we focused on EMs of SHR in the present study. The abundance of fluorescently labelled EMs in VSMCs confirmed the uptake of EMs by VSMCs. Some inhibitors related to different cellular‐uptake mechanisms including dynasore (Macia et al. 2006), CK‐666 (Sen et al. 2024), bafilomycin A1 (Wang et al. 2021) and cytochalasin D (Shoji et al. 2012) reduced the uptake of EMs, and attenuated VSMCs proliferation and migration induced by EMs of SHR. These findings indicate that the role of SHR's EMs was achieved through the uptake of EMs by VSMCs.

Proteomics analysis was performed to find the specific protein difference in EMs between WKY and SHR. Two significantly upregulated proteins, OPN and RISC, were preferentially screened out because of their close association with cell proliferation and migration (Gadeau et al. 1993; Lee et al. 2009; Vay et al. 2021). Both OPN and RISC contents in the EMs of SHR were much higher than these of WKY. The EMs from OPN knockdown‐treated VAFs of SHR lost their roles in promoting VSMCs proliferation and migration, but the EMs from RISC knockdown‐treated VAFs of SHR remained their roles. These results indicate that OPN in the EMs is responsible for the roles of SHR's EMs in stimulating cell proliferation and migration. Protein interaction analyses with STRING database predicted the interaction of OPN with other proteins. Among these proteins, the interaction of OPN with integrin αVβ3 and CD44 caught our attention because previous study showed that oral intake of milk OPN promoted intestinal proliferation and differentiation by OPN‐integrin αVβ3 and OPN‐CD44 signalling pathways (Jiang et al. 2023). Single‐cell sequencing data of human aortic cells in the Single Cell database showed that OPN, integrin αVβ3 and CD44 existed in VAFs and VSMCs. Molecular docking analyses with the Protein DataBank supported OPN binding to integrin αVβ3 and CD44. The roles of EMs in promoting VSMCs' proliferation and migration were almost abolished by specific blockade of integrin αVβ3, but were not significantly affected by CD44 knockdown. Co‐immunoprecipitation and SPR assay confirmed the binding of OPN to integrin αVβ3 in VSMCs. These findings indicate that the increased OPN in EMs of SHR was responsible for the roles of EMs in promoting VSMCs proliferation and migration in WKY and SHR. Evidence in the present study showed that FAK‐PI3K‐AKT signalling mediated the effects of EMs of SHR on VSMCs proliferation and migration via activating integrin αVβ3. The downstream PI3K/AKT pathway of integrin αVβ3 is supported by previous findings in several other cells (Isenovic et al. 2009; Li et al. 2024; Pérez‐Núñez et al. 2025). Moreover, the pathway is further supported by our findings in vivo.

There is a close interaction between vascular remodelling and hypertension (Humphrey 2021). In order to avoid the vascular remodelling secondary to blood pressure change, local OPN knockdown was used in the present study instead of systemic OPN knockdown. Knockdown of OPN in the carotid artery attenuated local vascular remodelling in SHR without significant effect on blood pressure, indicating that the increased vascular OPN expression in SHR is crucial for vascular remodelling and vascular OPN may be a key target for attenuation of vascular remodelling in hypertension. OPN is involved in a variety of pathophysiological processes, and milk OPN serves as a nutrient to play beneficial roles in human health and disease (Fleming et al. 2024; Sorensen and Christensen 2023). However, overdose of OPN may cause some toxicological and pathological effects, such as promoting cell proliferation, migration and vascular remodelling.

EMs of SHR promoted vascular remodelling and hypertension in both WKY and SHR. It is noted that the strong hypertensive effect appeared 3 weeks after application of the EMs. In the first week, EMs of SHR had no significant effect on blood pressure. These results suggest that the hypertensive effect may be secondary to vascular remodelling induced by the EMs of SHR. The increased vascular OPN content and phosphorylation of PI3K, AKT and FAK in the S‐EMs‐treated WKY and SHR may be responsible for the S‐EMs‐induced vascular remodelling, which was supported by the in vitro findings that the S‐EMs promotes VSMCs proliferation and migration via transferring OPN and subsequently activating integrin αVβ3/FAK/PI3K/AKT signalling pathway. Intervention of the EMs may be a therapeutic strategy for attenuation of vascular remodelling in hypertension.

EVs from WKY inhibited VSMCs proliferation and vascular remodelling of SHR via transferring miR‐155‐5p to inhibit angiotensin converting enzyme (ACE) transcription, while EVs from SHR promoted VSMCs proliferation and vascular remodelling through increased ACE and miR‐135‐5p transfer (Ye et al. 2022). EVs of WKY attenuated hypertension while EVs of SHR induced and aggravated hypertension (Ren et al. 2020). Similar to the roles of EVs from SHR, EMs of SHR also promoted VSMCs proliferation and migration, vascular remodelling and hypertension. However, EMs are different from EVs in that the effects of EMs from SHR were mediated by the increased OPN transfer and subsequent activation of integrin αVβ3/FAK/PI3K/AKT signalling pathway, and that EMs of WKY had no significant effects in attenuating VSMCs proliferation and migration, vascular remodelling and hypertension. It seems that EMs and EVs co‐regulate VSMCs phenotype and vascular remodelling with different molecular signalling mechanisms. Both EMs and EVs are important for intercellular communication between VAFs and VSMCs in vascular remodelling and hypertension.

A limitation of the present study is that the mechanism of OPN in the EMs acting on integrin αVβ3 is not determined. It is known that most of the roles of OPN at cellular level are attributed to extracellular effects of secreted OPN where receptor binding leads to activation of specific intracellular signalling pathways, and OPN interacts with integrin β3 through its Arg‐Gly‐Asp (RGD) sequence (Bastos et al. 2023). It is possible that the RGD region of the OPN is encapsulated in EMs and OPN in the EMs is unable to act on integrin αVβ3. We suppose that OPN dissociates from EMs in VSMCs after the uptake of EMs, and act on integrin αVβ3 to activate downstream signalling pathways.

In conclusion, EMs from VAFs of SHR promote VSMCs proliferation and migration of WKY and SHR via transferring OPN and the activation of integrin αVβ3/FAK/PI3K/AKT signalling pathway. Knockdown of OPN in carotid arteries attenuates local vascular remodelling in SHR. Repeated intravenous injections of the EMs of SHR promote vascular remodelling and hypertension. These results indicate the importance of EMs from VAFs of SHR in vascular remodelling in SHR. OPN in the EMs contributes to VSMC proliferation, migration and vascular remodelling via activation of integrin αVβ3/FAK/PI3K/AKT signalling pathway. Intervention of vascular OPN or EMs may be a potential therapeutic strategy for attenuation of vascular remodelling in hypertension.

## 4 Materials and Methods

### 4.1 Animals

Eight‐week‐old male WKY and SHR were obtained from Vital River Laboratory Animal Technology Co., Ltd (Beijing, China). Experiments were performed following the Guide for the Care and Use of Laboratory Animals (NIH, 8th edition) and approved by the Experimental Animal Care and Use Committee of Nanjing Medical University. The criterion for rats used in this study was that the SBP was higher than 150 mmHg in SHR, and lower than 140 mmHg in WKY. One WKY rat was excluded because of a SBP of approximately 140 mm Hg. Rats were housed in a 12/12 h light/dark cycling environment with controlled temperature and humidity, and free access to tap water and standard laboratory chow. The rat was euthanised with pentobarbital sodium (150 mg/kg, iv) at the end of the experiment.

### 4.2 Primary Cell Culture

VAFs and VSMCs were respectively isolated from the adventitia and media of thoracic aorta of WKY and SHR, and cultured as we described previously (Ren et al. 2020). The primary VAFs and VSMCs were identified as we reported recently (Ye et al. 2022). The primary cells from the 3rd to the 5th passages were used in this study.

### 4.3 Isolation of sEVs, EMs and SMs

sEVs, EMs and SMs were isolated from the medium of VAFs with ultracentrifugation method (Zhang et al. 2021). When the VAFs reached the 80% confluence, the cells were washed thrice with PBS and cultured in a serum‐free medium for 48 h. The medium was centrifuged at 300 × *g* for 10 min to remove cells, and the supernatant was centrifuged at 10,000 × *g* for 1 h to remove cellular debris. The supernatant was filtered through a 0.22 µm polyethersulfone filter (Thermo Fisher Scientific, Rockford, IL, USA) to reduce microparticle contamination, and then was subjected to ultracentrifugation at 167,000 × *g* for 4 h using a Type 45 Ti rotor (Beckman Coulter, Brea, CA, USA). The obtained bullet was EVs. Since the EVs were obtained through a 0.22 µm filter, which conformed more closely to sEVs according to recommended nomenclature (Welsh, Goberdhan, O'Driscoll, Buzas, et al. 2024; Welsh, Goberdhan, O'Driscoll, Théry, et al. 2024). Then, the supernatant was treated with ultracentrifugation using a Type 45 Ti rotor at 167,000 *g* for 16 h. The obtained bullet was EMs, and the supernatant was subjected to ultracentrifugation at 367,000 *g* for 16 h using a Beckman Coulter Type 70 Ti rotor (Beckman Coulter) for 16 h. The obtained bullet was SMs. All bullets of sEVs, EMs and SMs were dissolved in PBS containing 25 mM HEPES (pH 7.2), and were used directly or stored at 4°C for up to 2 days, or at –80°C for up to 3 months.

### 4.4 sEVs, EMs and SMs Quantification

The same volume of cell medium (70 mL) of each sample was collected for isolation of sEVs, EMs and SMs. The isolated pellet from each sample was suspended in 200 µL PBS. The suspension was mixed with same volume of RIPA Lysis Buffer (10 µL) for extracting protein. Total protein contents in sEVs, EMs and SMs were measured with Pierce BCA Protein Assay Kit (Beyotime Biotechnology, Shanghai, China). The sEVs, EMs and SMs contents were expressed as mg protein/mL, which were used as an index of the amount of sEVs, EMs and SMs (Zhang et al. 2021). After determining the dose‐effect, the concentration at 40 µg protein/mL was used to examine the effects of sEVs, EMs and SMs.

### 4.5 Atomic Force Microscope Imaging

Morphology of sEVs, EMs and SMs was examined with atomic force microscope (AFM) as previously reported (Zhang et al. 2021). The sample solution (20 µL) was diluted with PBS (20 µL) and incubated with (3‐aminopropyl) triethoxysilane (AP)‐modified mica substrates (Ted Pella Inc, Altadena, CA, USA) for 3 min. The substrates were washed twice with 50 µL PBS to remove unbound particles. Measurement was performed in PBS at room temperature using a Dimension FastScan microscope and ScanAsyst Fluid+tips (Bruker Instruments, Karlsruhe, BW, Germany) at a nominal radius of about 5 nm and spring constants of 0.7 N/m in an off‐resonance tapping mode. The images were taken at 0.75 Hz. The AFM diameter and height of sEVs, EMs and SMs were analysed with Gwyddion software (Version 2.64, http://www.gwyddion.net).

### 4.6 Transmission Electron Microscopy Imaging

The morphology of sEVs, EMs and SMs was further observed with transmission electron microscopy (TEM) (Zhang et al. 2021). Briefly, 20 µL of samples were loaded onto a carbon‐coated copper grid (Nisshin EM Corporation, Tokyo, Japan) for 5 min at 4°C, and then stained with 2% uranyl acetate and dried for 10 min. The grids were visualised with transmission electron microscope (Tecnai G2 Spirit TEM, Zeiss, Oberkochen, Germany).

### 4.7 Labelling and Uptake of EMs

EMs were labelled with a fluorescent dye Elab Fluor‐647 NHS ester (Elabscience, Wuhan, Hubei, China) following the manufacturer's instruction. The EMs solution was adjusted to a concentration of approximately 2 mg/mL with Labelling Buffer, and mixed well with same volume of Elab Fluor‐647 NHS ester (10 mg/mL) avoiding violent oscillation. After incubating in a 37°C constant temperature incubator in the dark for 30 min, the solution was transferred to the ultrafiltration core, and centrifuged at 12,000 *g* for 5 min, repeating four times to remove the unbound dye. Then, PBS was added for final ultrafiltration at 12,000 *g* for 5 min. The filter element was inverted and placed in a collection tube. After centrifuging at 12,000 *g* for 1 min, the supernatant was the solution of Elab Fluor‐647 labelled EMs. VSMCs were seeded on a 35‐mm dish in a DMEM culture medium at about 20,000 cells per well overnight, and then the cells were treated with the fluorescent‐labelled EMs in serum‐free DMEM medium. Images were acquired using confocal microscopy (LSM700, Carl Zeiss AG, Oberkochen, BW, Germany).

### 4.8 Proteomics and Proteomic Analysis

Proteomics was performed on VAFs‐derived EMs from 3 WKY and 3 SHR to show the protein difference between WKY and SHR. The EMs were lysed in RIPA buffers, and equal amounts of protein were run on a NuPAGE bis‐Tris gel. Liquid chromatography coupled with mass spectrometry (LC‐MS/MS) was used to analyse the peptide. Mass analysis of the peptide was performed using a Q Exactive Plus mass spectrometer (Thermo Fisher Scientific, Rockford, IL, USA) in positive ion mode. The data was acquired using a data‐dependent top 20 method dynamically choosing the most abundant precursor ions from the survey scan (300–1800 m/z) for higher energy collisional dissociation (HCD) fragmentation. The instrument was run with peptide recognition mode enabled. The original data of each sample were merged and retrieved using Scaffold 4.3.2 (Proteome Discoverer software, Thermo Fisher Scientific, Rockford, IL, USA), and the proteins were identified and quantitatively analysed. Hierarchical clustering analysis was performed using clustering software (Cluster 3.0, Institute of Medical Science, University of Tokyo, Japan) and open‐source Java Treeview software (Treeview 3.0, https://bitbucket.org/TreeView3Dev/treeview3/src) (Juan and Huang 2007). Stratified cluster analysis was systematically implemented to characterise differentially expressed proteins between the WKY and SHR cohorts. The heatmap configuration included vertical columns corresponding to the samples annotated along the x‐axis, and horizontal rows representing individual protein entities labelled on the y‐axis. Protein expression levels were normalised using Z‐score transformation, with colourimetric gradation reflecting relative expression intensities. Red and blue hues indicate significant upregulation and downregulation, respectively. Grey areas signify undetectable protein quantities. The volcano plot was made with Volcanoplot APP (https://www.originlab.com). Protein sequences are searched using the InterProScan software to identify protein domain signatures from the InterPro member database Pfam (Quevillon et al. 2005). Enrichment analysis was applied based on the Fisher’ exact test, considering the whole quantified proteins as the background dataset. Benjamini–Hochberg correction for multiple testing was used to adjust derived *p* values. Only functional categories and pathways with *p* values under a threshold of 0.05 were considered significant.

### 4.9 Construction of the PPI Network

The STRING database (STRING11.0, http://string‐db.org) was used to assess protein‐protein interactions (PPIs) in functional protein association networks using core factors as query proteins (Szklarczyk et al. 2023). OPN gene Spp1 entered the database to observe its interaction with other proteins. Furthermore, integrin β3 gene Itgb3 and PI3K gene Pik3r1 entered the database to find their interaction with other proteins. Required confidence (combined score) > 0.1 was used as the cut‐off criterion.

### 4.10 Protein‐Protein Docking

The 3D structure of OPN (AF‐P10451), CD44 (AF‐P26051), integrin αV (AF‐P06756), integrin β3 (AF‐P05106) and FAK (AF‐Q05397) were obtained from the Alphafold https://alphafold.com/). According to experimental requirements, molecular docking of full‐length or fragment proteins was performed using Alphafold 3. During the docking process, the protein structures were converted to a PDB file, which contained all the polar residues with hydrogen. Protein and protein docking adopted the GRAMM semi‐flexible docking method, and the two related proteins were uploaded to the third‐party website (https://gramm.compbio.ku.edu/request) for semi‐flexible protein docking to form the conformation with the lowest binding energy (Singh et al. 2024). The models of the complex were analysed using LigPlot^+^ (Version: 2.2; https://www.ebi.ac.uk/thornton‐srv/software/LigPlus) and PyMOL (Version: 1.5.6; https://pymol.org/).

### 4.11 Single Nucleus RNA‐Seq Data Collection and Analyses

Aortic‐single nucleus RNA‐seq data were obtained from three human individuals in the Single Cell portal database of the Broad Institute (https://singlecell.broadinstitute.org/single_cell). Literature confirmed that the data were publicly available (Pirruccello et al. 2022). The data were used to analyse the cell types and gene expression levels in the aorta. Uniform manifold approximation and projection (UMAP) revealed 12 main clusters and the expressions of OPN, integrin β_3_ and CD44. Each dot represents an individual nucleus, coloured and labelled by putative cell type as identified from Leiden clustering. The colour depth of the dot represents the gene expression level. Curated meta‐information and processing steps about each dataset were elaborated in the database.

### 4.12 VAFs With OPN or RISC Knockdown

Lentiviral vectors targeting OPN and RISC (shOPN and shRISC) were constructed and identified by Genechem (Shanghai, China). The lentiviral vector with the EGFP tag was hU6‐MCS‐CMV‐EGFP‐Puro. This vector expresses enhanced green fluorescent protein (EGFP) and puromycin resistance genes while interfering with the target gene. The sequences of shRNA were listed in Table S1. A designed shRNA‐interfering fragment was constructed downstream of the U6 promoter in a lentiviral vector using molecular biological methods. The purified lentivirus was used to infect the target cells, and puromycin was used for screening to obtain stable cell lines expressing shRNA. VAFs of SHR were treated with shOPN (MOI = 80), shRISC (MOI = 80), NC or PBS for 24 h. The medium was refreshed and cultured for 48 h. EGFP fluorescence expressed by the lentivirus in the VAFs was examined under a fluorescence microscope (Axio Vert. Al, Zeiss, Germany) to evaluate the lentiviral infection efficiency. OPN and RISC mRNA and protein expressions were examined with qPCR and WB to evaluate the efficiency of OPN or RISC knockdown. The OPN or RISC knockdown‐treated VAFs were cultured and used for isolating EMs to determine whether OPN or RISC in the EMs contributes to VSMCs' proliferation and migration.

### 4.13 CD44 Knockdown in VSMCs

CD44 siRNAs (si‐CD44) and negative control were commercially obtained from GenScript (Nanjing, Jiangsu, China). The sequences of si‐CD44 were listed in Table S1. VSMCs were transfected with si‐CD44 (50 nM) using the Lipofectamine 3000 kit (Invitrogen, Carlsbad, CA, USA) for 48 h.

### 4.14 Phalloidin Staining

F‐actin in VSMCs was revealed by Phalloidin staining (Wang et al. 2023). The cells were fixed with 4% paraformaldehyde for 15 min, and treated with 0.5% TritonX‐100/PBS solution for 5 min. Then, the cells were incubated with Phalloidin‐TRITC (100 nM, Yeasen, Shanghai, China) for 1 h, and DAPI for 5 min in the dark at 37°C. Images were captured under laser scanning confocal microscopy (Carl Zeiss AG, Oberkochen, BW, Germany).

### 4.15 Surface Plasmon Resonance

Surface plasmon resonance (SPR) was used to detect the binding affinities of OPN to integrin αVβ3 with a Biacore X100 SPR system (Cytiva, Uppsala, Sweden). Integrin αVβ3 was immobilised on the flow channel of a CM5 sensor chip (GE Healthcare, Chicago, IL, USA) with the standard amine coupling method. For immobilisation, integrin αVβ3 was diluted in double‐distilled water and loaded onto the chip after activation of the sensor surface with (3‐dimethylaminopropyl)‐3‐ethylcarbodiimide (EDC)‐N‐hydroxysuccinimide (NHS). The unoccupied surface area was blocked with ethanolamine. The running buffer was 10 mM HEPES, 150 mM NaCl, 0.05% n‐Octyl‐β‐D‐glucopyranoside, pH 7.5. OPN protein in a series of increasing concentrations was applied to the channels as an analyte at a flow rate of 30 µL/min. A blank immobilisation was performed for correcting the binding response. Sensorgram was made with Biacore T200 evaluation software (Version 2.0.3). Response units (RU) were obtained during the equilibration phase at each concentration for steady‐state affinity fittings.

### 4.16 Evaluation of VSMCs Proliferation

Cell counting kit‐8 (CCK8) kit and 5‐ethynyl‐2′‐deoxyuridine (EdU) incorporation assay were used to evaluate VSMCs proliferation as we previously reported (Sun et al. 2017). CCK8 kits were commercially obtained from Beyotime Institute of Biotechnology (Shanghai, China). The absorbance was measured at 450 nm with a microplate reader (ELX800, BioTek, Vermont, USA). EdU incorporation assay was performed with Cell‐LightTM EdU Apollo594 In vitro Imaging Kits (RiboBio, Guangzhou, Guangdong, China). The images were captured by a fluorescence microscope (Carl Zeiss Meditec, Jena, Germany). EdU‐positive cells were counted and normalised by the number of total cells stained with Hoechst‐33342.

### 4.17 Evaluation of VSMCs Migration

VSMCs migration was evaluated by Boyden Chamber Assay and Wound‐Healing Assay as we previously reported (Wu et al. 2021). For Boyden Chamber Assay, the cells that had moved to the lower surface of the membrane were stained with crystal violet. The average number of stained cells in five randomly selected fields was counted. For Wound‐Healing Assay, the wound healing was photographed and calculated at 0 and 24 h with inverted microscope (MF53, Micro‐shot Technology Co, Guangzhou, Guangdong, China), and the average migrated distance was calculated.

### 4.18 Quantitative Real‐Time PCR

A two‐step qPCR approach was employed to quantitatively analyse mRNA expression levels. The data were analysed with StepOne Plus software (version 2.3, Applied Biosystems). The relative values were determined with the ΔΔCt method. GAPDH was used as an internal control. The sequence of primers for qRT‐PCR was listed in Table S2.

### 4.19 Western Blotting

VAFs, VSMCs, carotid artery, aorta and mesenteric artery were homogenised in lysis buffer. Supernatant was extracted for measuring total protein. The same amounts of total protein were separated with 10% SDS‐PAGE and transferred to a PVDF membrane. The membranes were blocked with 5% milk and incubated with the first antibody overnight at 4°C. Then, a secondary antibody conjugated with HRP was added and incubated for 1 h at room temperature. GAPDH was used as an internal control. The information on antibodies was listed in Table S3.

### 4.20 Immunofluorescence Staining

Immunofluorescence staining for vimentin and α‐SMA was performed in VAFs and VSMCs, respectively. Immunofluorescence staining for OPN was performed in carotid artery, aorta and mesenteric artery sections. The samples were fixed with 4% paraformaldehyde for 15 min, 0.2% Triton X‐100 for 5 min and 10% bovine albumin for 1 h. Then the cells were incubated with the first antibody overnight at 4°C, followed by secondary Alexa Fluor 594 or 488 antibody at 1:1000 dilution in PBS at room temperature for 1 h in the dark. The nuclei were stained with DAPI at 1:1000 dilution for 5 min. Staining was visualised using a fluorescence microscope (Axio Vert. Al, Zeiss, Germany).

### 4.21 Co‐Immunoprecipitation

Co‐immunoprecipitation was performed to show the OPN‐integrin αVβ3 interaction in VSMCs. The cells were harvested and lysed using RIPA Lysis Buffer supplemented with cocktail inhibitors (MedChem Express, Monmouth Junction, NJ, USA). Cell lysates were centrifuged at 12,000 *g* for 15 min at 4°C. Supernatants were taken 40 µL (4% of the total lysate quantity) as the input group, the remaining supernatants were incubated with 2 µg integrin αVβ3 antibody (Santa Cruz Biotechnology, Santa Cruz, CA, USA) or control IgG overnight at 4°C, then incubated with protein A/G beads (Santa Cruz Biotechnology, Santa Cruz, CA, USA) at 4°C for 6 h. After the incubation, the bead‐protein complexes were washed three times with RIPA buffer to remove non‐specifically bound proteins and mixed with SDS‐PAGE Sample Loading Buffer (5×) boiled at 95°C for 5 min. The supernatants were collected for subsequent immunoblotting analysis with anti‐integrin αVβ3 and anti‐OPN antibodies.

### 4.22 Knockdown of OPN in Carotid Artery of Rats

Adeno‐associated virus (AAV) delivery of shRNA was used for knockdown of OPN in carotid artery of rats. The AAV‐shOPN and scrambled sequence (negative control, NC) were commercially obtained from Genechem (Shanghai, China). The sequence targeting OPN is the same as the siRNA sequence targeting OPN used in vivo (Table S1). Pluronic F127 gel is commonly used for limiting chemical or virus diffusion (Zheng et al. 2024). AAV‐shOPN (7 × 10^12^ v.g/mL, 10 µL) or NC was added to 60 µL of 30% pluronic F127 gel (Sigma‐Aldrich, St. Louis, MO, USA). Bilateral carotid arteries were separated from the adjacent tissue using parafilm. The gel was dropped on the surface of the carotid artery three times at an interval of 10 min, and the wound was closed.

### 4.23 Masson's Staining

The carotid artery, aorta and mesenteric artery were prefixed with paraformaldehyde and were embedded with paraffin. Sections were stained with Masson's trichrome staining. Images were captured under light microscope (BX‐51, Olympus, Tokyo, Japan). Media thickness, lumen diameter and the media thickness ratio to lumen diameter were used to evaluate vascular remodelling.

### 4.24 Measurement of Blood Pressure and Sympathetic Activity

Systolic blood pressure of tail artery was measured in the conscious state with a non‐invasive computerised tail‐cuff system (NIBP, ADInstruments, Sydney, New South Wales, Australia). Mean arterial pressure was measured under anaesthesia via femoral artery intubation. Renal sympathetic nerve activities (RSNA) and blood pressure were recorded with a PowerLab system with data acquisition software (8SP, ADInstruments Inc.) as we previously reported (Ye et al. 2020).

### 4.25 Chemicals

Dynasore, CK‐666, AKTi1/2, LY294002, puromycin, bafilomycin A1, cytochalasin D, cyclo(‐RGDfK), Y15, OPN and integrin αVβ3 were purchased from MedChemExpress (Monmouth Junction, NJ, USA). Collagenase II was obtained from Gibco (Carlsbad, CA, USA).

### 4.26 Statistical Analysis

Experiments were performed in a randomised and double‐blinded manner. Data are shown as mean ± SD. Power analysis was performed to ensure adequate sample size. Data was fit to a normal distribution. Unpaired Student's *t*‐test was performed to compare the differences between two groups. One‐way or two‐way ANOVA followed by Bonferroni test was used for multiple comparisons. Repeated measure ANOVA was used for the analysis of dose‐effect and time‐effect. *p* < 0.05 was considered statistically significant.

## Author Contributions

**Jing‐Xiao Wang**: Conceptualization (equal); data curation (lead); formal analysis (lead); funding acquisition (supporting); investigation (lead); methodology (lead); writing ‐ original draft (lead). **Xiao‐Yu Xu**: Data curation (equal); formal analysis (equal); investigation (equal); methodology (supporting). **Hong‐Ke Dong**: Data curation (supporting); investigation (supporting); methodology (supporting). **Yi‐Ming Wang**: Data curation (supporting); investigation (supporting); methodology (supporting). **Min Dai**: Formal analysis (supporting); investigation (supporting); methodology (supporting). **Bing Zhou**: Funding acquisition (supporting); methodology (supporting); writing ‐ review and editing (supporting). **Yue‐Hua Li**: Conceptyalization; writing‐review and editing (supporting). **Guo‐Qing Zhu**: Conceptualization (lead); data curation (lead); formal analysis (lead); funding acquisition (lead); methodology (lead); project administration (lead); resources (lead); supervision (lead); validation (lead); visualization (lead); writing ‐ original draft (lead); writing ‐ review and editing (lead). **Xiao‐Qing Xiong**: Conceptualization (lead); formal analysis (lead); funding acquisition (lead); methodology (supporting); project administration (supporting); supervision (supporting); validation (supporting); visualization (supporting); writing ‐ original draft (supporting); writing ‐ review and editing (supporting).

## Conflicts of Interest

The authors declare no conflicts of interest.

## Supporting information




**Supplementary Materials**: jev270146‐sup‐0001‐SuppMat.docx

## Data Availability

All data supporting the findings of this study are available from the corresponding author on reasonable request.
