# Supplementary material for: Exomeres From Adventitial Fibroblasts of Spontaneously Hypertensive Rats Promote Vascular Remodelling via Transferring Osteopontin
Source: J Extracell Vesicles. 2025 Aug 6;14(8):e70146. doi: 10.1002/jev2.70146 (PMC12326188; doi:10.1002/jev2.70146)
Supplement: Supplementary file 1 — Supplementary Materials: jev270146‐sup‐0001‐SuppMat.docx [file JEV2-14-e70146-s001.docx]

**Online Supplementary Data**

**Exomeres from adventitial fibroblasts of spontaneously hypertensive rats promote vascular remodeling via transferring osteopontin**

**Wang JX, et al.**

**(8 supplementary tables and 3 supplementary tables)**

**
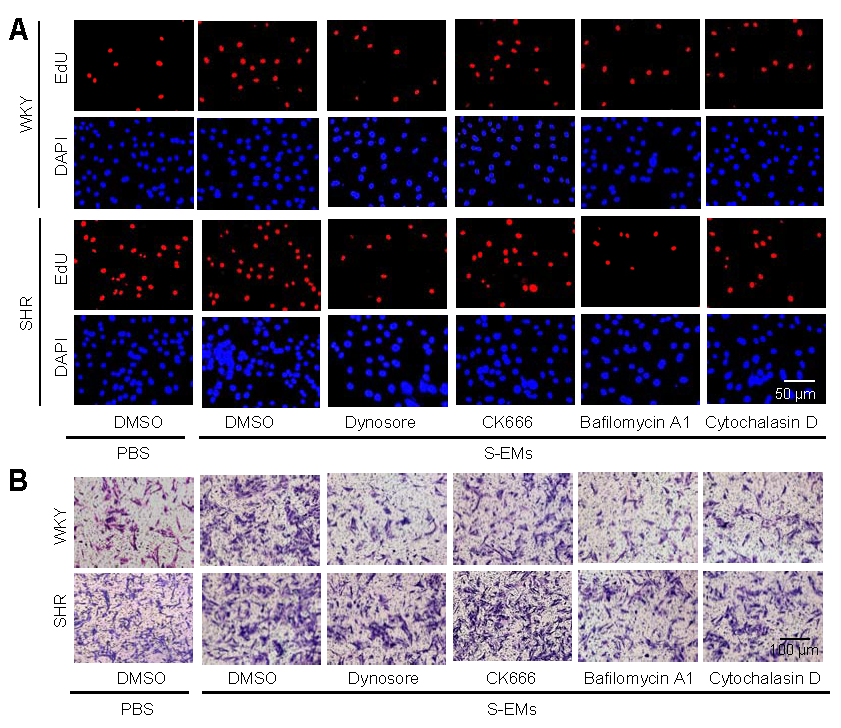
**

**Figure S1** Representative images showing the effects of dynosore, CK666, bafilomycin A1 and cytochalasin D on the S-EMs-induced VSMCs proliferation and migration of WKY and SHR. The cells were pre-incubated with 1% DMSO (vehicle), dynosore (20 µM), CK666 (25 µM), bafilomycin A1 (100nM) or cytochalasin D (5 µM) for 30 min, and then incubated with S-EMs for 24 h. The VSMCs proliferation and migration were evaluated with EdU-positive cells and Boyden chamber assay, respectively.


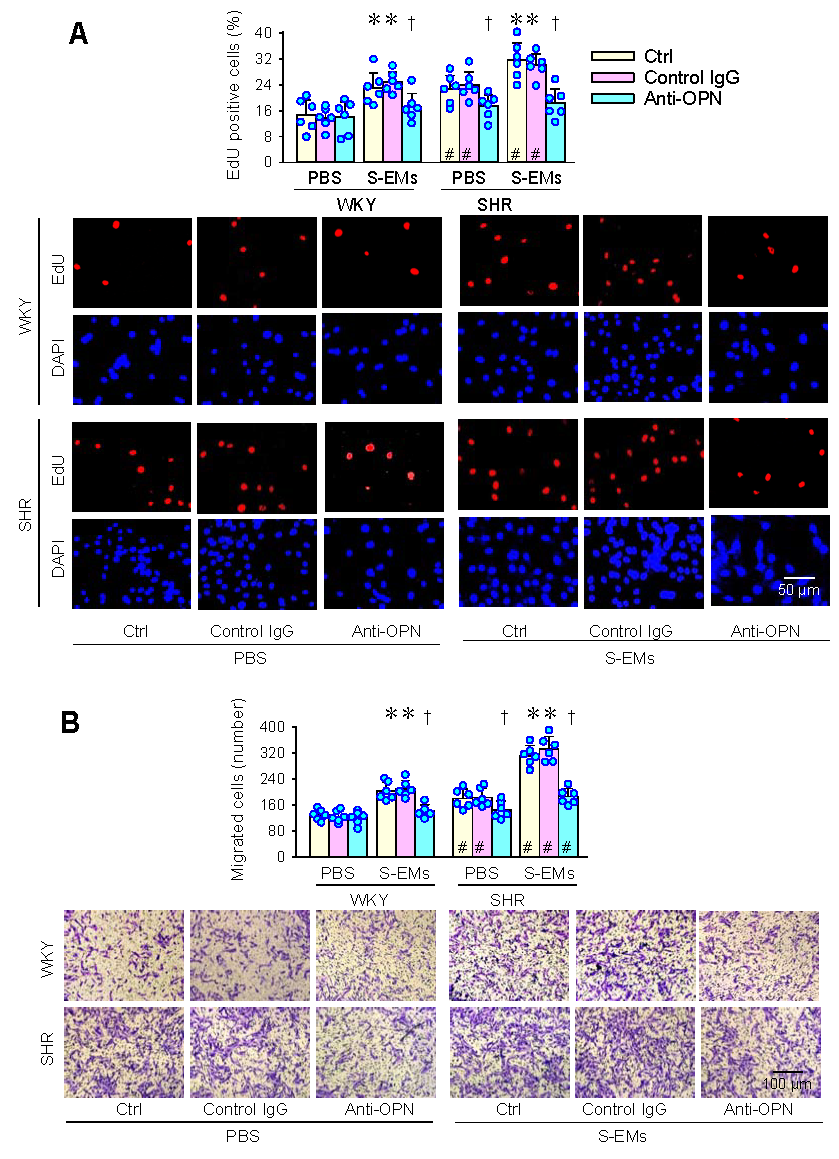


**Figure S2** Effects of OPN antibody on the S-EMs-induced VSMCs proliferation and migration of WKY and SHR. OPN antibody (Anti-OPN, 10 μg/mL) were administrated 2 h before S-EMs (40 μg/mL). PBS (Ctrl) and control IgG were used as controls. A, cell proliferation were evaluated by the percentage of EdU-positive cells. B, cell migration were evaluated by Boyden chamber assay. Values are mean±SD. *P<0.05 vs PBS; †P<0.05 vs Ctrl or Control IgG; #P<0.05 vs WKY. n=6. Two-way ANOVA followed by Bonferroni test.


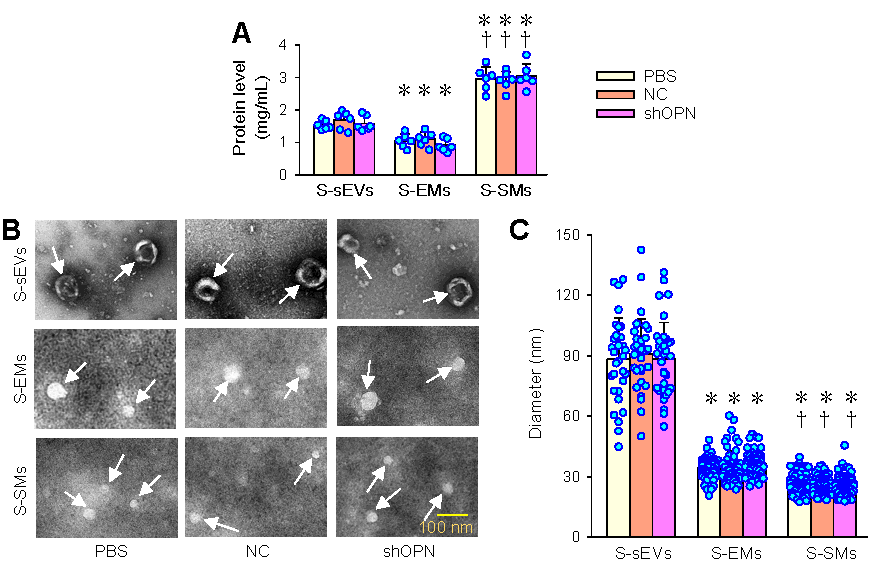
**Figure S3** Effects of OPN knockdown in VAFs of SHR on the VAFs-released S-sEVs, S-EMs and S-SMs. VAFs from SHR were infected with OPN shRNA encoded-lentiviral vector (shOPN, MOI=10) for knockdown of OPN. PBS and negative control shRNA encoded-lentiviral vector (NC) were used as control. A, protein levels in S-sEVs, S-EMs and S-SMs. B, representative images of transmission electron microscope (TEM) showing the nanoparticles. C, nanoparticle diameter according to TEM images from 6 independent samples. Values are mean±SD. *P<0.05 vs S-sEVs; †P<0.05 vs S-EMs. n=6. Two-way ANOVA followed by Bonferroni test.


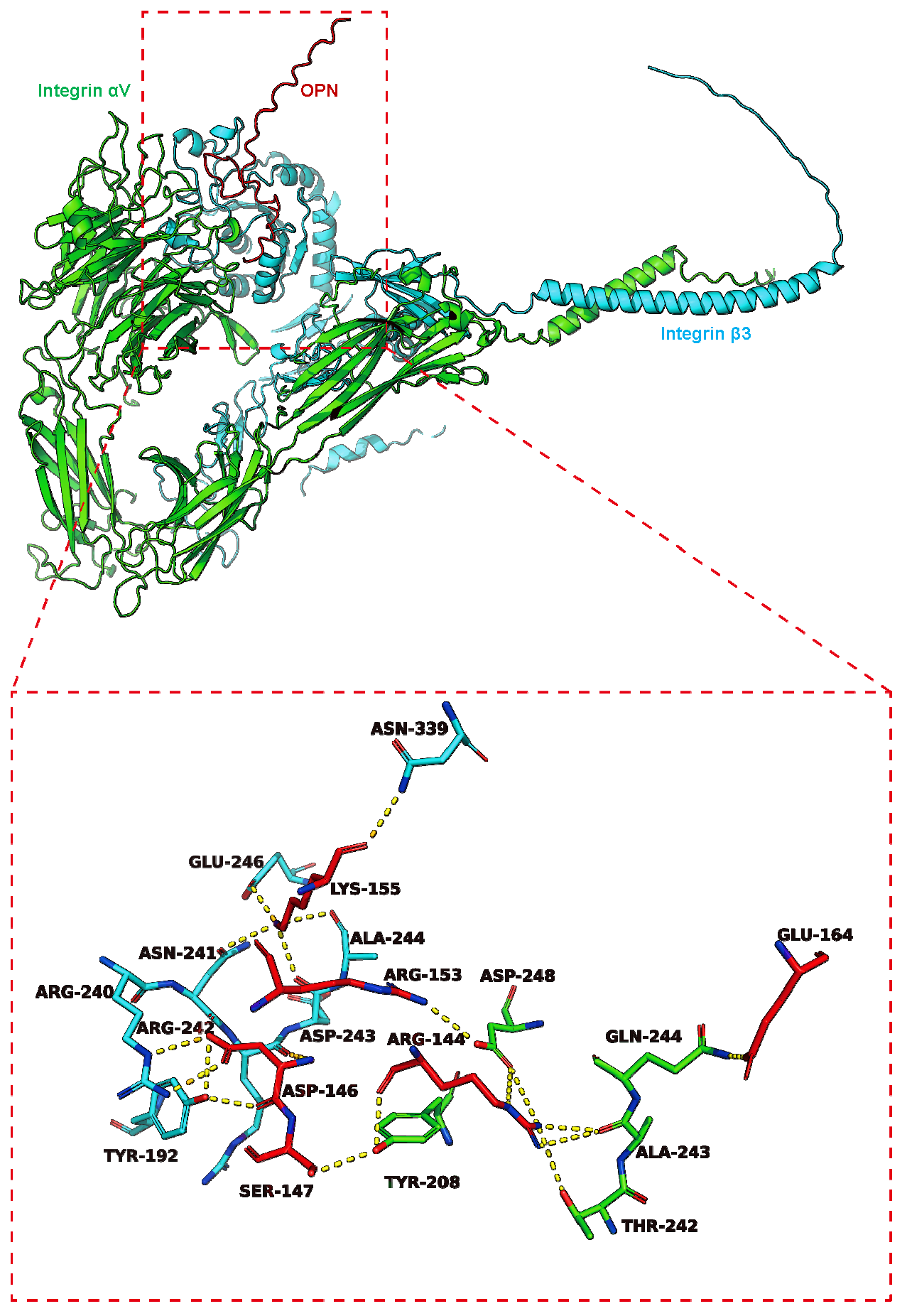
**Figure S4** Molecular docking of OPN (red), integrin αV (green), and integrin β3 (blue).

**
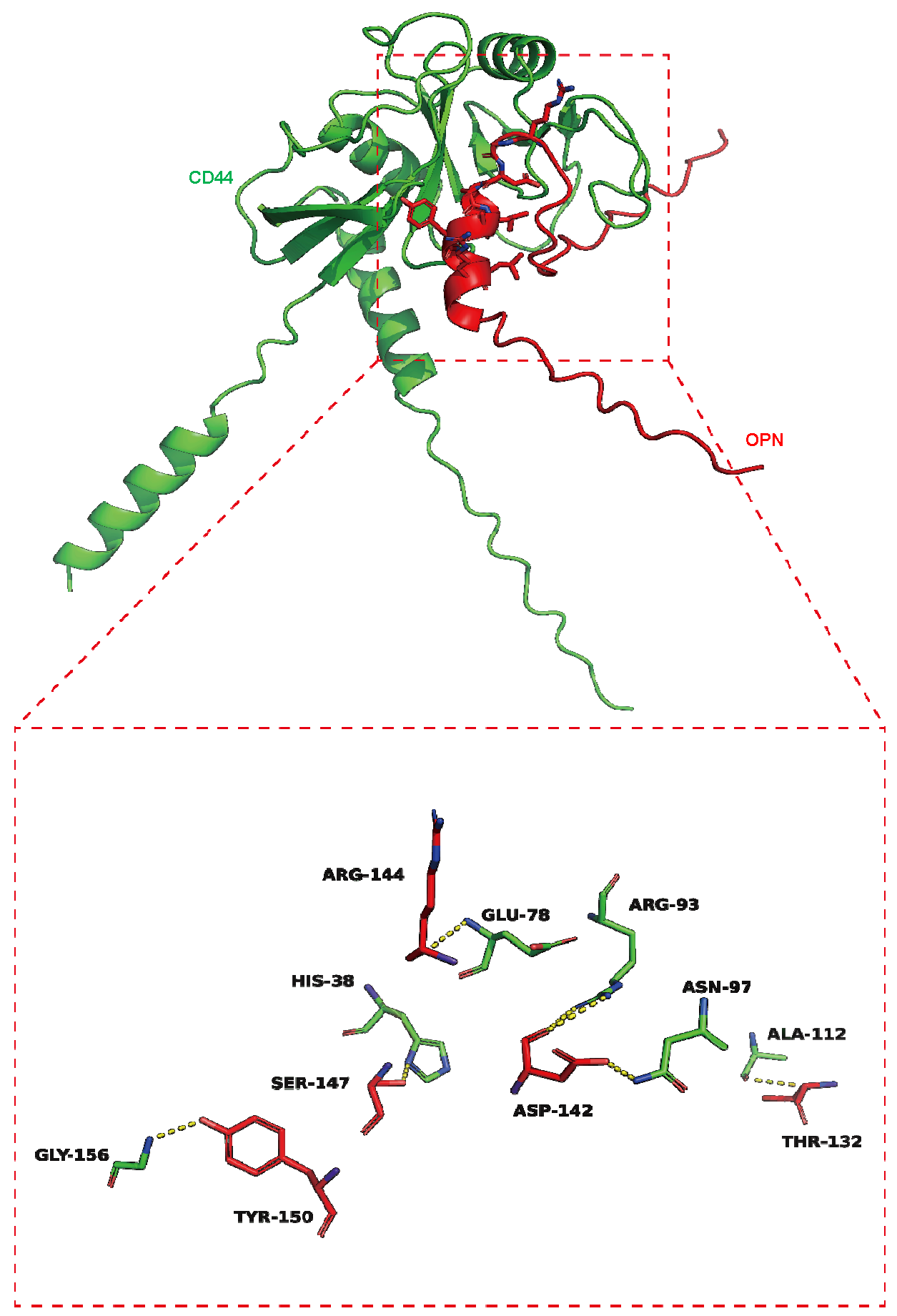
Figure S5** Molecular docking of OPN (red) and CD44 (green).

**
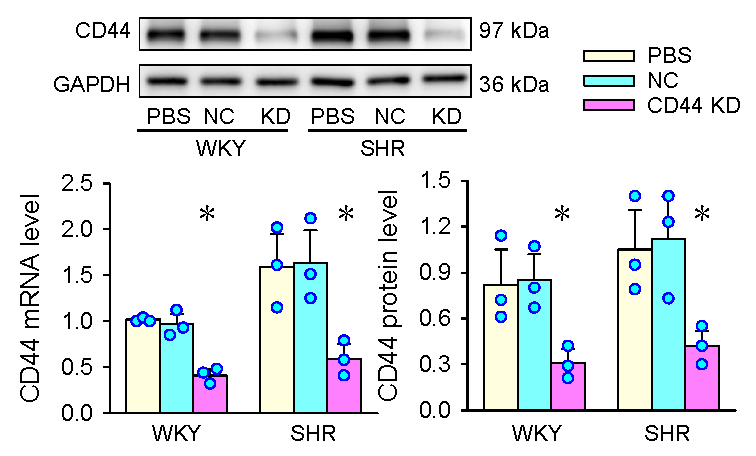
Figure S6** Effects of CD44 knockdown (KD) on the relative value of CD44 mRNA and protein expressions in VSMCs of WKY and SHR. si-CD44 (50 nM) was used for CD44 knockdown. Values are mean±SD. *P<0.05 vs PBS or NC. n=3. Two-way ANOVA followed by Bonferroni test.


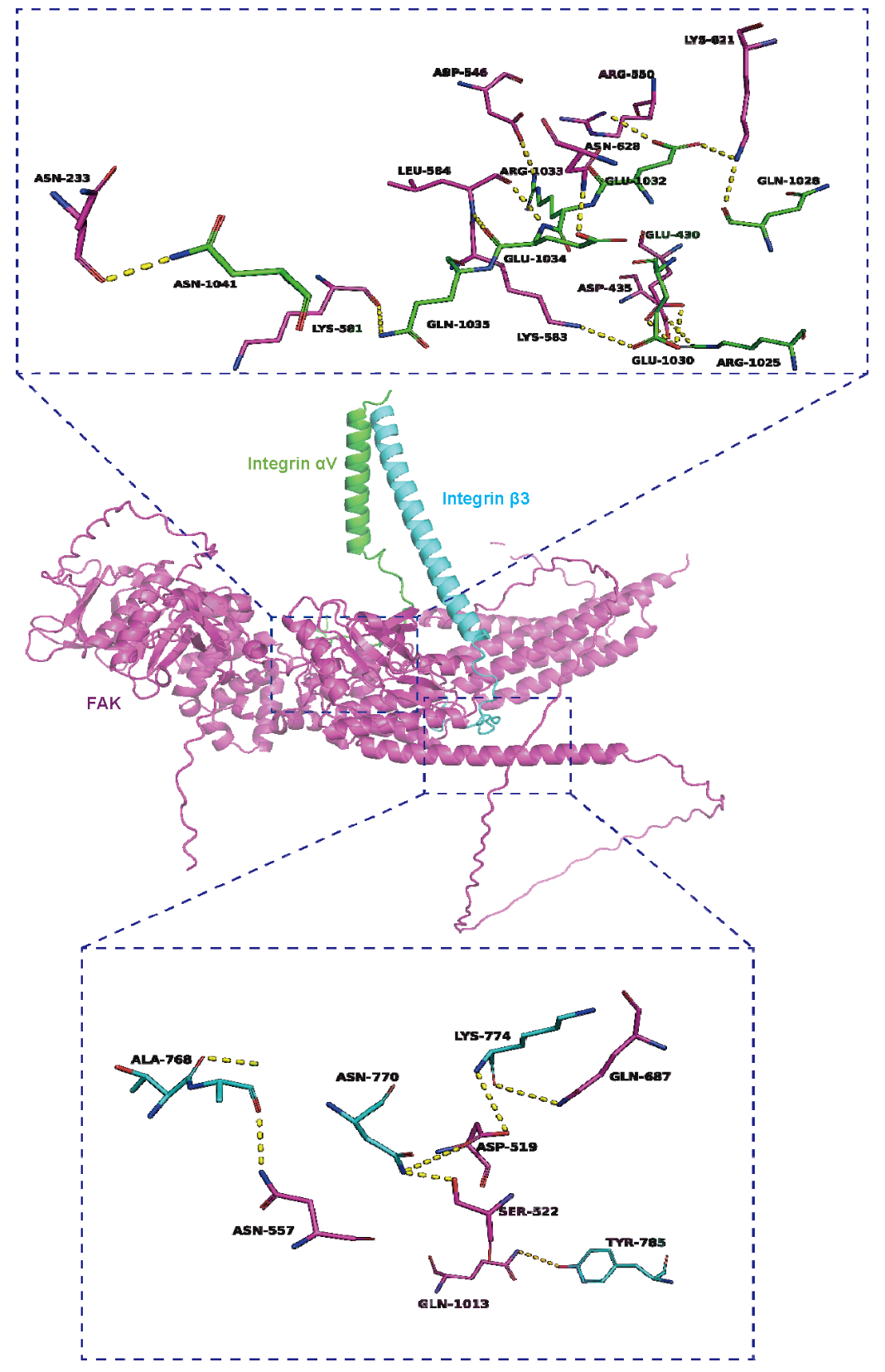


**Figure S7** Molecular docking of FAK (purple), integrin αV (green), and integrin β3 (blue).


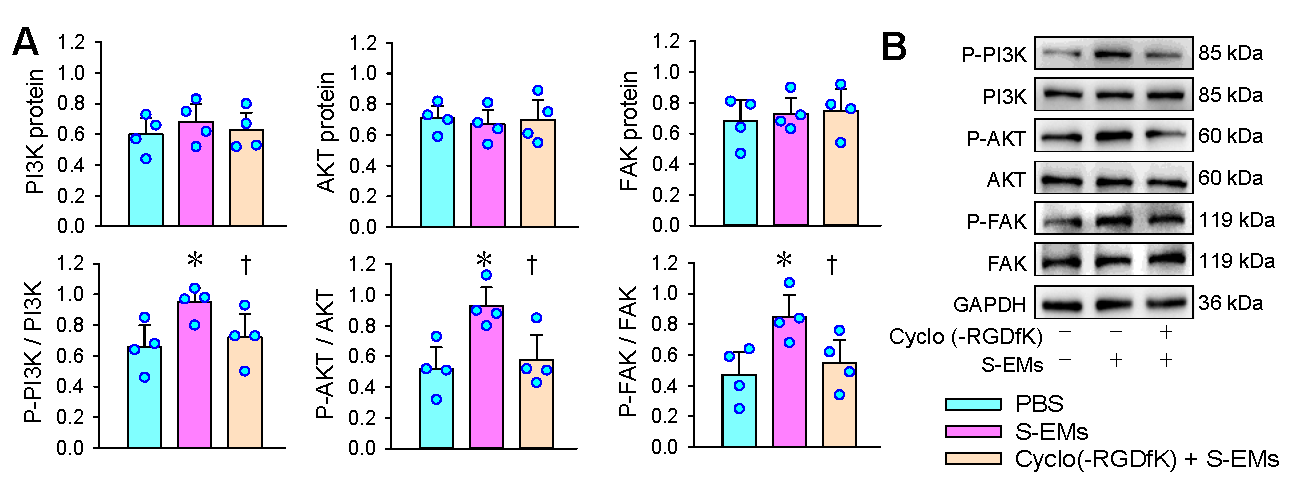


**Figure S8** Effects of integrin αVβ3 inhibitor Cyclo (-RGDfK) (10 mg/kg, ip, every 2 days, 10 times) on the roles of S-EMs (200 μg, iv, every 2 days, 10 times) in promoting the phosphorylation of PI3K, AKT and FAK in aorta of WKY. A, PI3K, AKT and FAK protein expressions and their phosphorylation levels in aorta. B, representative Western blot images. Values are mean±SD. *P<0.05 vs PBS; †P<0.05 vs S-EMs alone. n=4. One-way ANOVA followed by Bonferroni test.

**Table S1. shRNA sequence and siRNA sequence**

| Gene |  | Sequence |
| --- | --- | --- |
| OPN | Sense | 5’-AAGAUGAUAGGUAUCUGAAAU-3’ |
|  | Antisense | 5’-AUUUCAGAUACCUAUCAUCUU-3’ |
| RISC | Sense | 5’-ACACCUACAAGGAGACGCCUUAAGUCA-3’ |
|  | Antisense | 5’-UGACUUAAGGCGUCUCCUUGUAGGUGU-3’ |
| CD44 | Sense | 5’-GUUUGAAACAUGCAGGUAUUU-3’ |
|  | Antisense | 5’-AUACCUGCAUGUUUCAAACCC-3’ |

| **Table S2. Primers for qRT-PCR** | | |  |
| --- | --- | --- | --- |
| Name | Primer | Sequence |  |
| OPN | Forward | 5’-AGACTGGCAGTGGTTTGCTT-3’ |  |
|  | Reverse | 5’-ATGGCTTTCATTGGAGTTGC-3’ |  |
| RISC | Forward | 5’-GAGCTCTCGCGGCGGATCTGTCTCGTGC-3’ |  |
|  | Reverse | 5’-CTCCTGCTTGGTCACCAGCTTCATCATCT-3’ |  |
| CD44 | Forward | 5’-ACCATTGAGAAGAGCACCCC-3’ |  |
|  | Reverse | 5’-CACAGTTGAGGCAATGGTGG-3’ |  |
| GAPDH | Forward | 5’-TTCCAGGAGCGAGATCCCGCTAAC-3’ |  |
|  | Reverse | 5’-TTCAGGTGAGCCCCAGCCTTCT-3’ |  |

**Table S3. Antibodies for Western blot (WB) and immunofluorescence analyses (IF)**

| Antibody | Cat No | Company | Address | Dilution |
| --- | --- | --- | --- | --- |
| AGO2 | 2897T | CST | Beverly, MA, USA | 1:1000 (WB) |
| Alexa Fluor 488 | A-11059 | Thermo Fisher | Rockford, IL, USA | 1:1000 (IF) |
| Alexa Fluor 594 | A-11012 | Thermo Fisher | Rockford, IL, USA | 1:1000 (IF) |
| AKT | ab179463 | Abcam | Cambridge, MA, USA | 1:1000 (WB) |
| calnexin | ab22595 | Abcam | Cambridge, MA, USA | 1:1000 (WB) |
| CD44 | 37259T | CST | Beverly, MA, USA | 1:1000 (WB) |
| CD63 | Sc-5275 | Santa Cruz | Santa Cruz, CA, USA | 1:1000 (WB) |
| CD9 | ab172730 | Abcam | Cambridge, MA, USA | 1:500 (WB) |
| FAK | T55464S | Abmart Inc. | Shanghai, China | 1:1000 (WB) |
| GAPDH | 2118S | CST | Beverly, MA, USA | 1:1000 (WB) |
| integrin αVβ3 | sc-7312 | Santa Cruz | Santa Cruz, CA, USA | 1:1000 (WB) |
| OPN | 22952-1-AP | ProteinTech | Wuhan, China | 1:1000 (WB) |
| PCNA | ab92552 | Abcam | Cambridge, MA, USA | 1:1000 (WB) |
| PCSK9 | ab315480 | Abcam | Cambridge, MA, USA | 1:1000 (WB) |
| PI3K | T40115F | Abmart Inc. | Shanghai, China | 1:1000 (WB) |
| P-AKT(ser473) | ab81283 | Abcam | Cambridge, MA, USA | 1:1000 (WB) |
| P-FAK | T55587S | Abmart Inc. | Shanghai, China | 1:1000 (WB) |
| P-PI3K (Tyr199) | T40116F | Abmart Inc. | Shanghai, China | 1:1000 (WB) |
| RISC | YN3708 | ImmunoWay | Plano, TX, USA | 1:1000 (WB) |
| syntenin-1 | A5497 | Abclonal | Wuhan, China | 1:1000 (WB) |
| TGFBI | 3711S | CST | Beverly, MA, USA | 1:1000 (WB) |
| TSG101 | ab133586 | Abcam | Cambridge, MA, USA | 1:500 (WB) |
| vimentin | ET1610-39 | HUABIO | Hangzhou, China | 1:50 (IF) |
| α-SMA | ET1607-53 | HUABIO | Hangzhou, China | 1:50 (IF) |
| β-actin | 4967S | CST | Beverly, MA, USA | 1:1000 (WB) |
